# Supplementary material for: The structural basis of the genetic code: amino acid recognition by aminoacyl-tRNA synthetases
Source: Sci Rep. 2020 Jul 28;10:12647. doi: 10.1038/s41598-020-69100-0 (PMC7387524; doi:10.1038/s41598-020-69100-0)
Supplement: Supplementary file 1 — Supplementary Information. [file 41598_2020_69100_MOESM1_ESM.pdf]

Supplementary Information for Manuscript

The Structural Basis of the Genetic Code: Amino Acid Recognition by Aminoacyl-tRNA Synthetases

Florian Kaiser[a,b,*] Sarah Krautwurst[c], Sebastian Salentin[a], V. Joachim Haupt[a,b], Christoph Leberecht[c], Sebastian Bittrich[c], Dirk Labudde[c], Michael Schroeder[a]

[a] Biotechnology Center (BIOTEC), TU Dresden, Dresden, 01307, Germany, [b] PharmAI GmbH, Tatzberg 47, 01307 Dresden, Germany, [c] University of Applied Sciences Mittweida, Mittweida, 09648, Germany

*florian.kaiser@tu-dresden.de

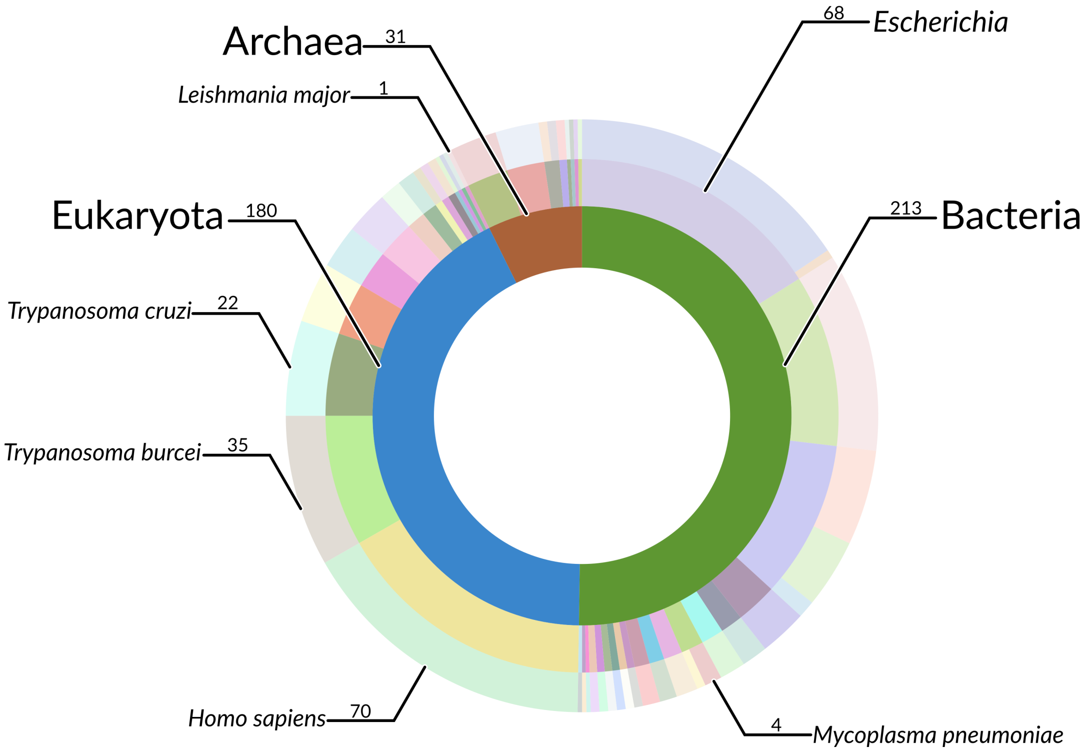

Fig. S1. Sunburst diagram of protein chains containing a catalytic Aminoacyl-tRNA synthetase (aaRS) domain co-crystallized with their amino acid ligand in respect to source species. The dataset (1) covers all three superkingdoms, contains human and structures of pathogenic species.

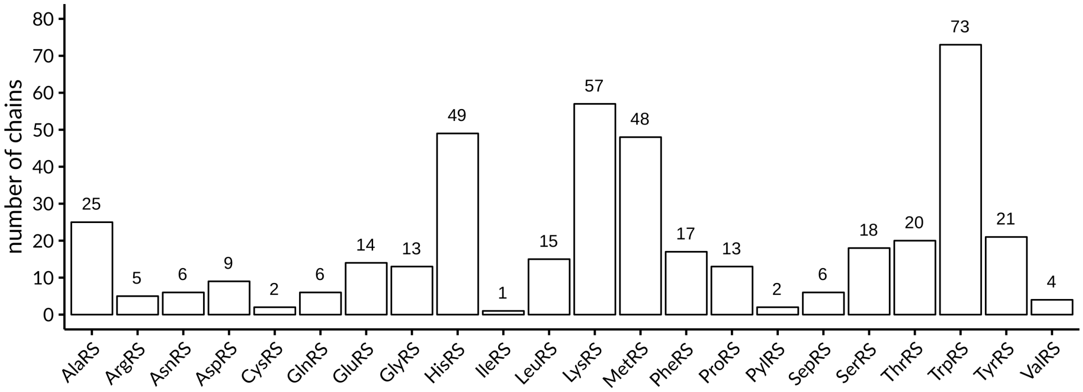

Fig. S2. The number of protein chains containing a catalytic aaRS domain for each of the 22 aaRSs. The dataset(1) used in this study contains structures for all aaRSs.

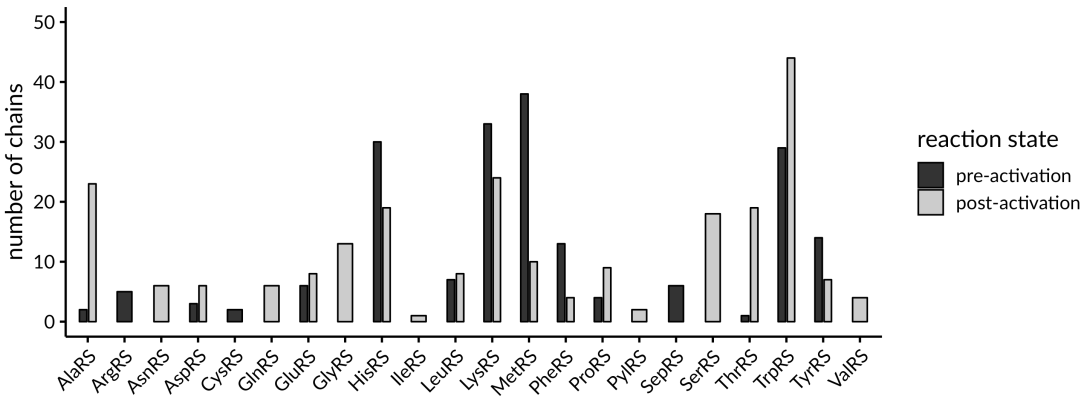

Fig. S3. The number of protein chains containing either an amino acid ligand (pre-activation) or an aminoacyl ligand (post-activation). For twelve aaRSs data was available for both reaction states.

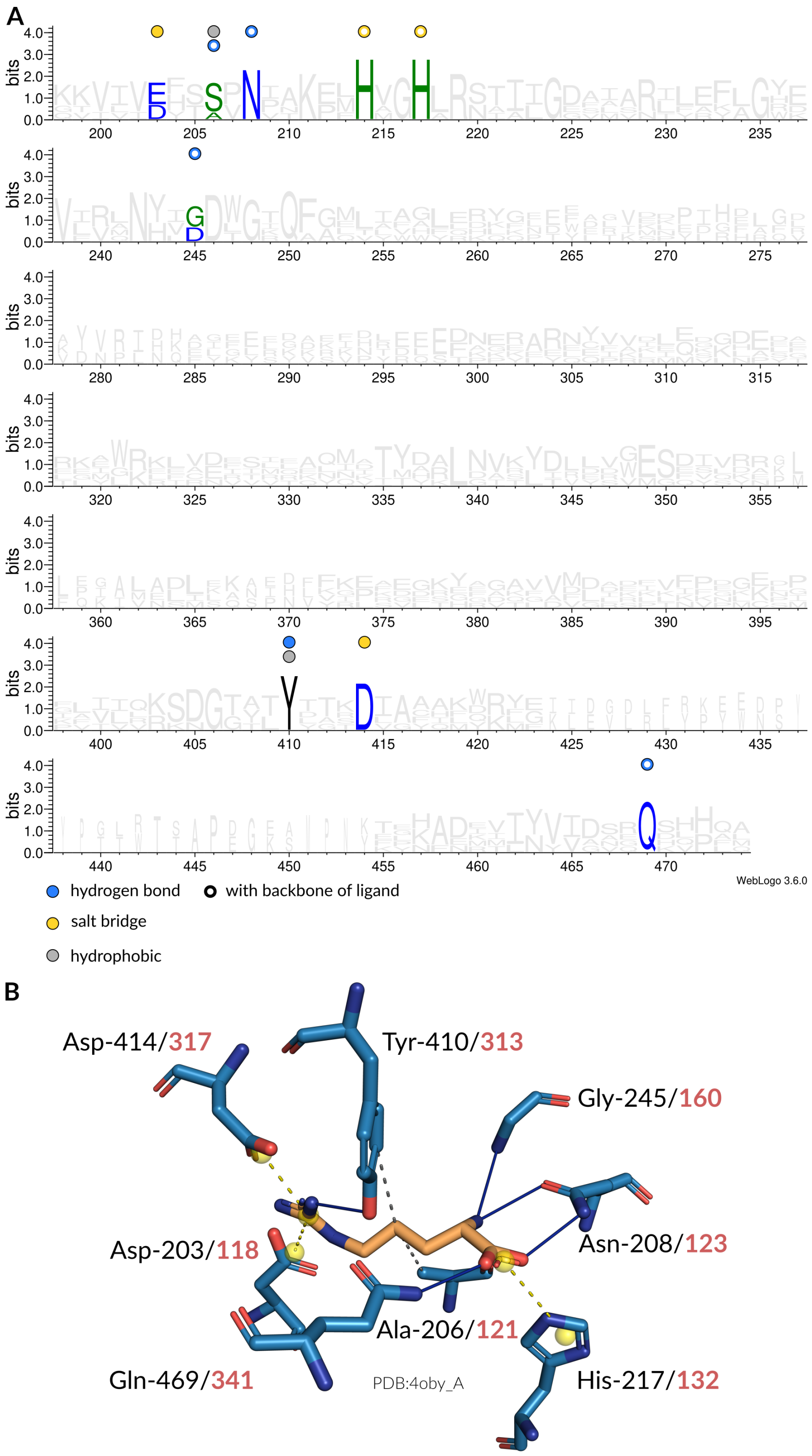

Fig. S4. Interaction patterns of arginyl-tRNA synthetase (ArgRS). (**A**) Sequence logo (2) of representative sequences for ArgRSs. Non-covalent interactions with the amino acid ligand occurring at certain positions are indicated by colored circles. Filled circles are interactions with the side chain atoms, while hollow circles are interactions with any of the backbone atoms of the amino acid ligand. (**B**) Depiction of interactions in the binding site (blue stick model) of an ArgRS from *Escherichia coli* (PDB:4oby chain A) with its ligand (orange stick model). Here, hydrogen bonds (solid blue lines), salt bridges (dashed yellow lines) and hydrophobic interactions (dashed gray lines) are established. The sequence positions of the interacting residues are given in accordance to the multiple sequence alignment (MSA) (black) as well as the original structure (red).

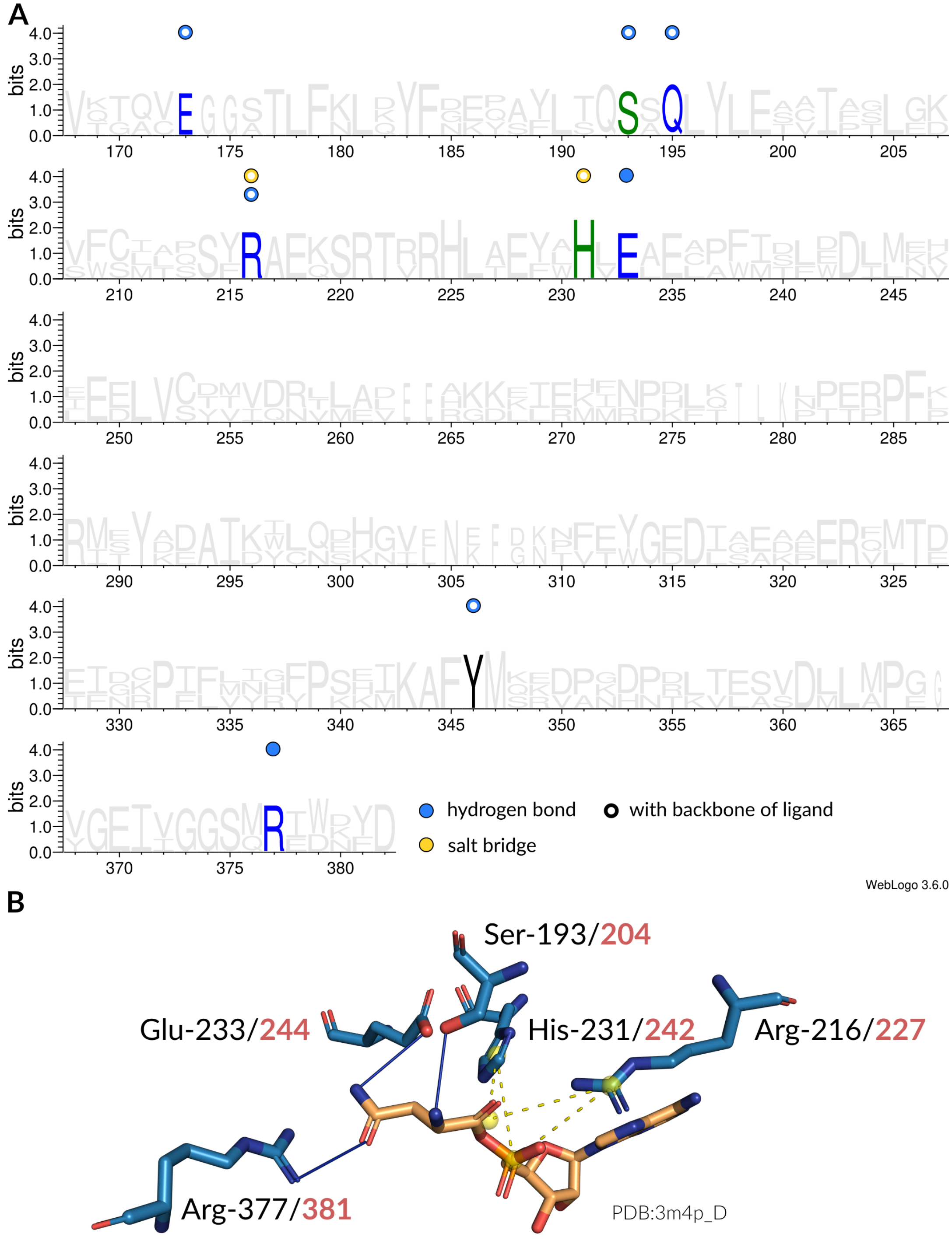

Fig. S5. Interaction patterns of asparaginyl-tRNA synthetase (AsnRS). (**A**) Sequence logo (2) of representative sequences for AsnRSs. Non-covalent interactions with the amino acid ligand occurring at certain positions are indicated by colored circles. Filled circles are interactions with the side chain atoms, while hollow circles are interactions with any of the backbone atoms of the amino acid ligand. (**B**) Depiction of interactions in the binding site (blue stick model) of an AsnRS from *Entamoeba histolytica* (PDB:3m4p chain D) with its ligand (orange stick model). Here, hydrogen bonds (solid blue lines), salt bridges (dashed yellow lines), and hydrophobic interactions (dashed gray lines) are established. The sequence positions of the interacting residues are given in accordance to the MSA (black) as well as the original structure (red).

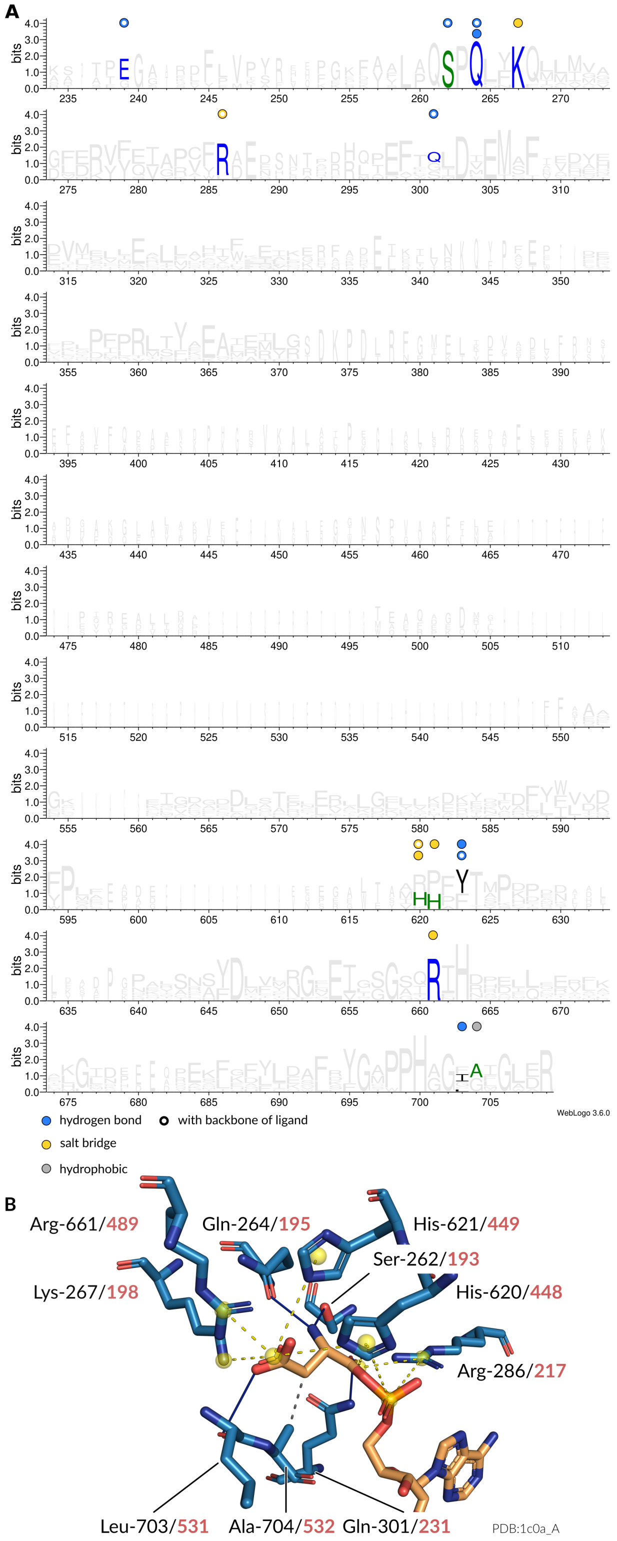

Fig. S6. Interaction patterns of aspartyl-tRNA synthetase (AspRS). (**A**) Sequence logo (2) of representative sequences for AspRSs. Non-covalent interactions with the amino acid ligand occurring at certain positions are indicated by colored circles. Filled circles are interactions with the side chain atoms, while hollow circles are interactions with any of the backbone atoms of the amino acid ligand. (**B**) Depiction of interactions in the binding site (blue stick model) of an AspRS from *Escherichia coli* (PDB:1c0a chain A) with its ligand (orange stick model). Here, hydrogen bonds (solid blue lines), salt bridges (dashed yellow lines), and hydrophobic interactions (dashed gray lines) are established. The sequence positions of the interacting residues are given in accordance to the MSA (black) as well as the original structure (red).

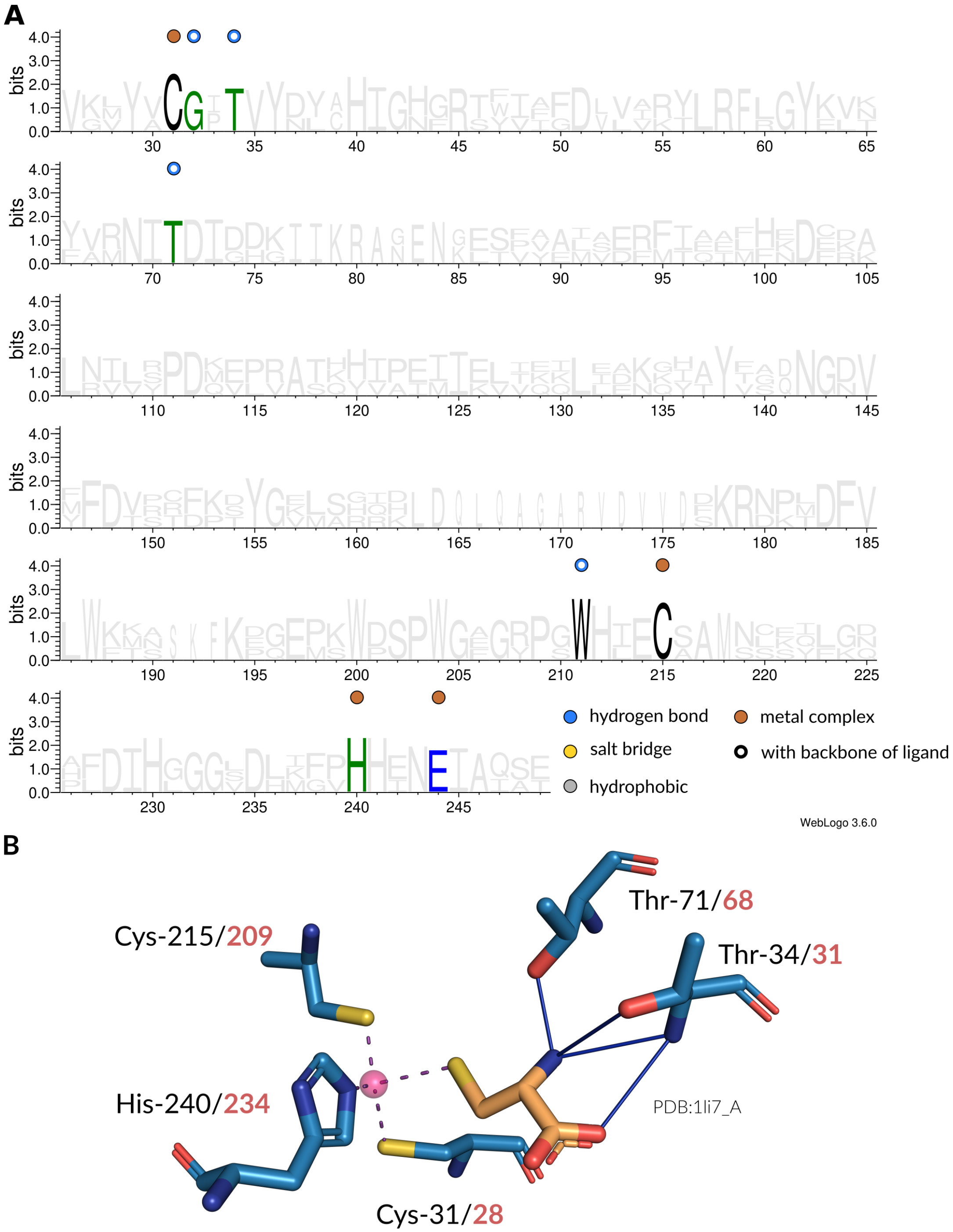

Fig. S7. Interaction patterns of cysteinyl-tRNA synthetase (CysRS). (**A**) Sequence logo (2) of representative sequences for CysRSs. Non-covalent interactions with the amino acid ligand occurring at certain positions are indicated by colored circles. Filled circles are interactions with the side chain atoms, while hollow circles are interactions with any of the backbone atoms of the amino acid ligand. (**B**) Depiction of interactions in the binding site (blue stick model) of an CysRS from *Escherichia coli* (PDB:1li7 chain A) with its ligand (orange stick model). Here, hydrogen bonds (solid blue lines) and metal complex interactions (dashed magenta lines) are established. The sequence positions of the interacting residues are given in accordance to the MSA (black) as well as the original structure (red).

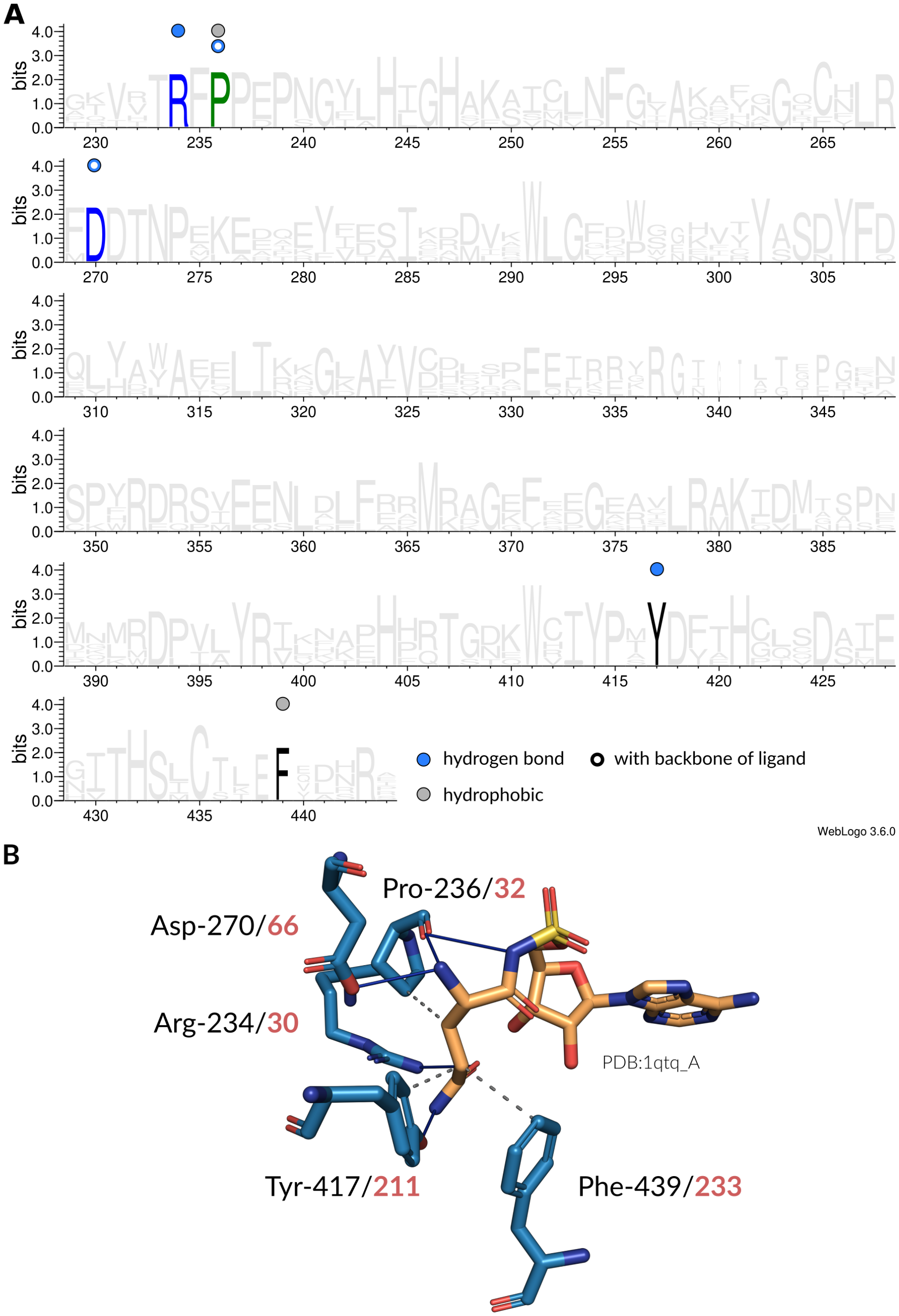

Fig. S8. Interaction patterns of glutaminyl-tRNA synthetase (GlnRS). (**A**) Sequence logo (2) of representative sequences for GlnRSs. Non-covalent interactions with the amino acid ligand occurring at certain positions are indicated by colored circles. Filled circles are interactions with the side chain atoms, while hollow circles are interactions with any of the backbone atoms of the amino acid ligand. (**B**) Depiction of interactions in the binding site (blue stick model) of an GlnRS from *Escherichia coli* (PDB:1qtq chain A) with its ligand (orange stick model). Here, hydrogen bonds (solid blue lines) and hydrophobic interactions (dashed gray lines) are established. The sequence positions of the interacting residues are given in accordance to the MSA (black) as well as the original structure (red).

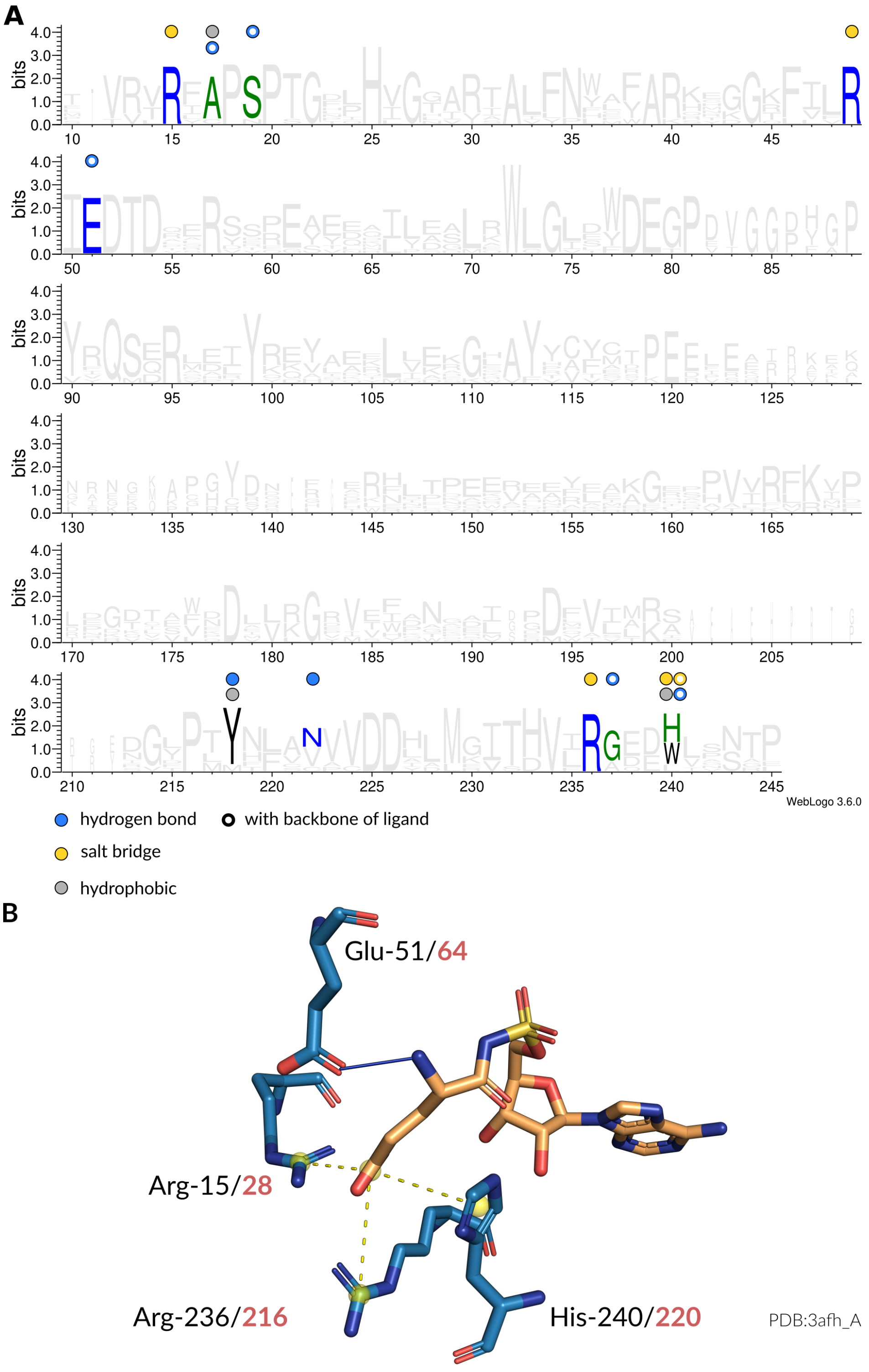

Fig. S9. Interaction patterns of glutamyl-tRNA synthetase (GluRS). (**A**) Sequence logo (2) of representative sequences for GluRSs. Non-covalent interactions with the amino acid ligand occurring at certain positions are indicated by colored circles. Filled circles are interactions with the side chain atoms, while hollow circles are interactions with any of the backbone atoms of the amino acid ligand. (**B**) Depiction of interactions in the binding site (blue stick model) of an GluRS from *Thermotoga maritima* (PDB:3afh chain A) with its ligand (orange stick model). Here, hydrogen bonds (solid blue lines) and salt bridges (dashed yellow lines) are established. The sequence positions of the interacting residues are given in accordance to the MSA (black) as well as the original structure (red).

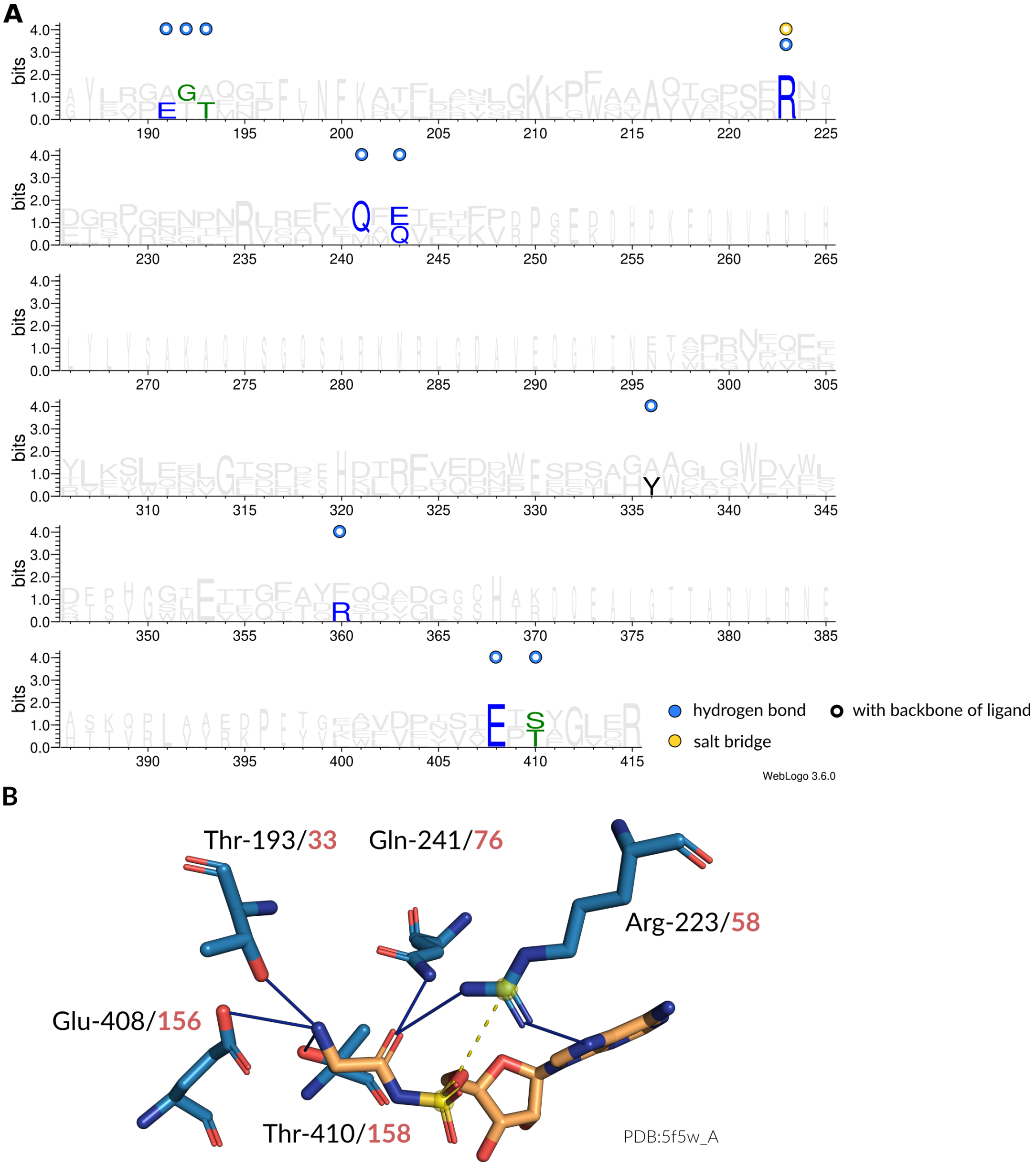

Fig. S10. Interaction patterns of glycyl-tRNA synthetase (GlyRS). (**A**) Sequence logo (2) of representative sequences for GlyRSs. Non-covalent interactions with the amino acid ligand occurring at certain positions are indicated by colored circles. Filled circles are interactions with the side chain atoms, while hollow circles are interactions with any of the backbone atoms of the amino acid ligand. (**B**) Depiction of interactions in the binding site (blue stick model) of an GlyRS from *Aquifex aeolicus* (PDB:5f5w chain A) with its ligand (orange stick model). Here, hydrogen bonds (solid blue lines) and salt bridges (dashed yellow lines) are established. The sequence positions of the interacting residues are given in accordance to the MSA (black) as well as the original structure (red).

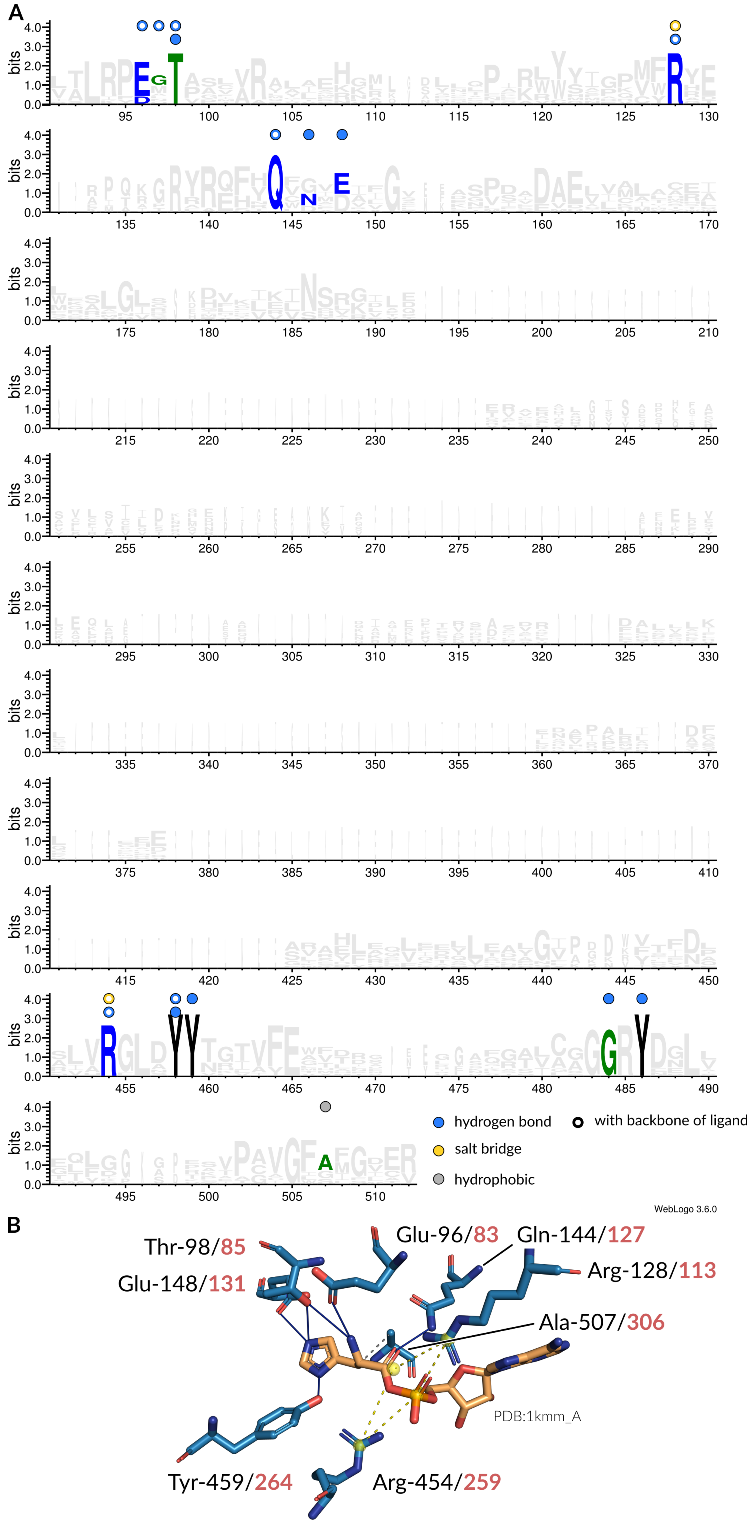

Fig. S11. Interaction patterns of histidyl-tRNA synthetase (HisRS). (**A**) Sequence logo (2) of representative sequences for HisRSs. Non-covalent interactions with the amino acid ligand occurring at certain positions are indicated by colored circles. Filled circles are interactions with the side chain atoms, while hollow circles are interactions with any of the backbone atoms of the amino acid ligand. (**B**) Depiction of interactions in the binding site (blue stick model) of an HisRS from *Escherichia coli* (PDB:1kmm chain A) with its ligand (orange stick model). Here, hydrogen bonds (solid blue lines), salt bridges (dashed yellow lines), and hydrophobic interactions (dashed gray lines) are established. The sequence positions of the interacting residues are given in accordance to the MSA (black) as well as the original structure (red).

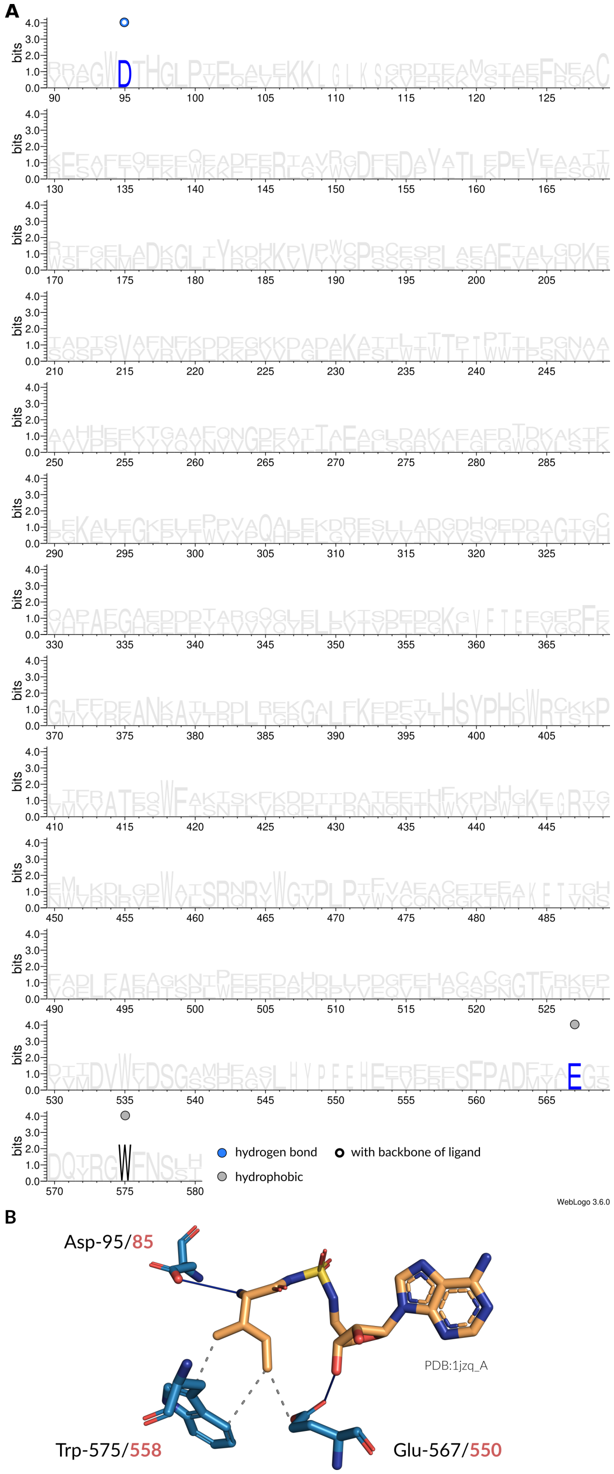

Fig. S12. Interaction patterns of isoleucyl-tRNA synthetase (IleRS). (**A**) Sequence logo (2) of representative sequences for IleRSs. Non-covalent interactions with the amino acid ligand occurring at certain positions are indicated by colored circles. Filled circles are interactions with the side chain atoms, while hollow circles are interactions with any of the backbone atoms of the amino acid ligand. (**B**) Depiction of interactions in the binding site (blue stick model) of an IleRS from *Thermus thermophilus* (PDB:1jzq chain A) with its ligand (orange stick model). Here, hydrogen bonds (solid blue lines) and hydrophobic interactions (dashed gray lines) are established. The sequence positions of the interacting residues are given in accordance to the MSA (black) as well as the original structure (red).

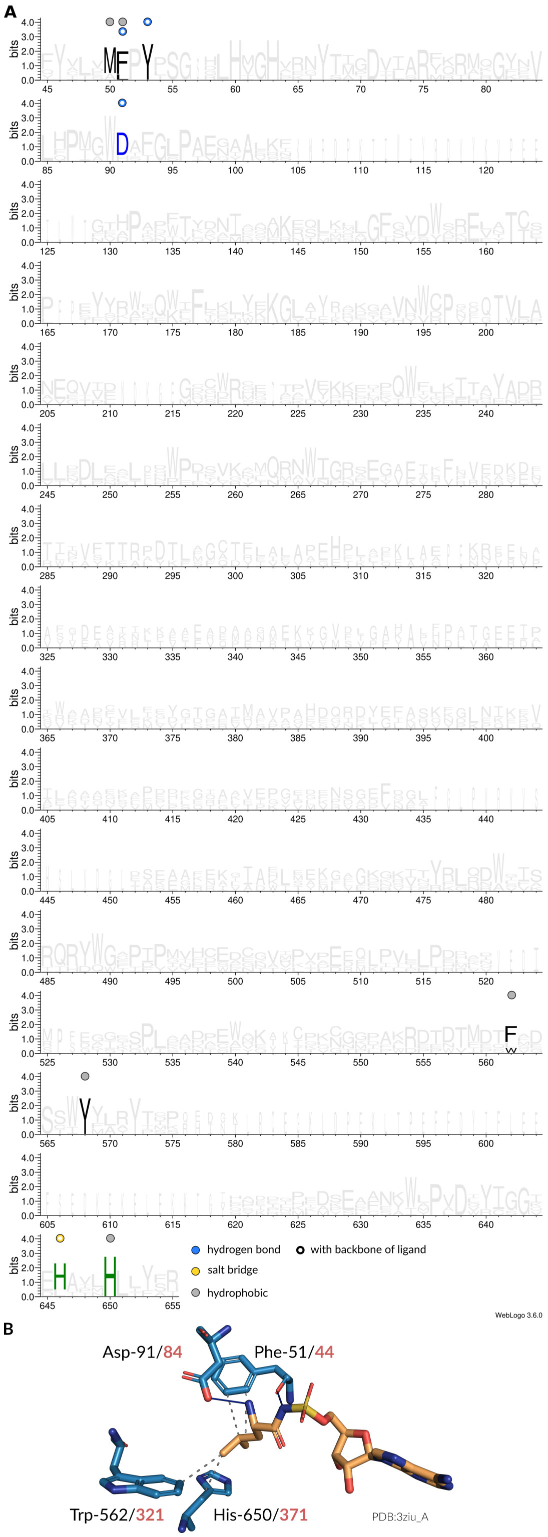

Fig. S13. Interaction patterns of leucyl-tRNA synthetase (LeuRS). (**A**) Sequence logo (2) of representative sequences for LeuRSs. Non-covalent interactions with the amino acid ligand occurring at certain positions are indicated by colored circles. Filled circles are interactions with the side chain atoms, while hollow circles are interactions with any of the backbone atoms of the amino acid ligand. (**B**) Depiction of interactions in the binding site (blue stick model) of an LeuRS from *Mycoplasma mobile* (PDB:3ziu chain A) with its ligand (orange stick model). Here, hydrogen bonds (solid blue lines) and hydrophobic interactions (dashed gray lines) are established. The sequence positions of the interacting residues are given in accordance to the MSA (black) as well as the original structure (red).

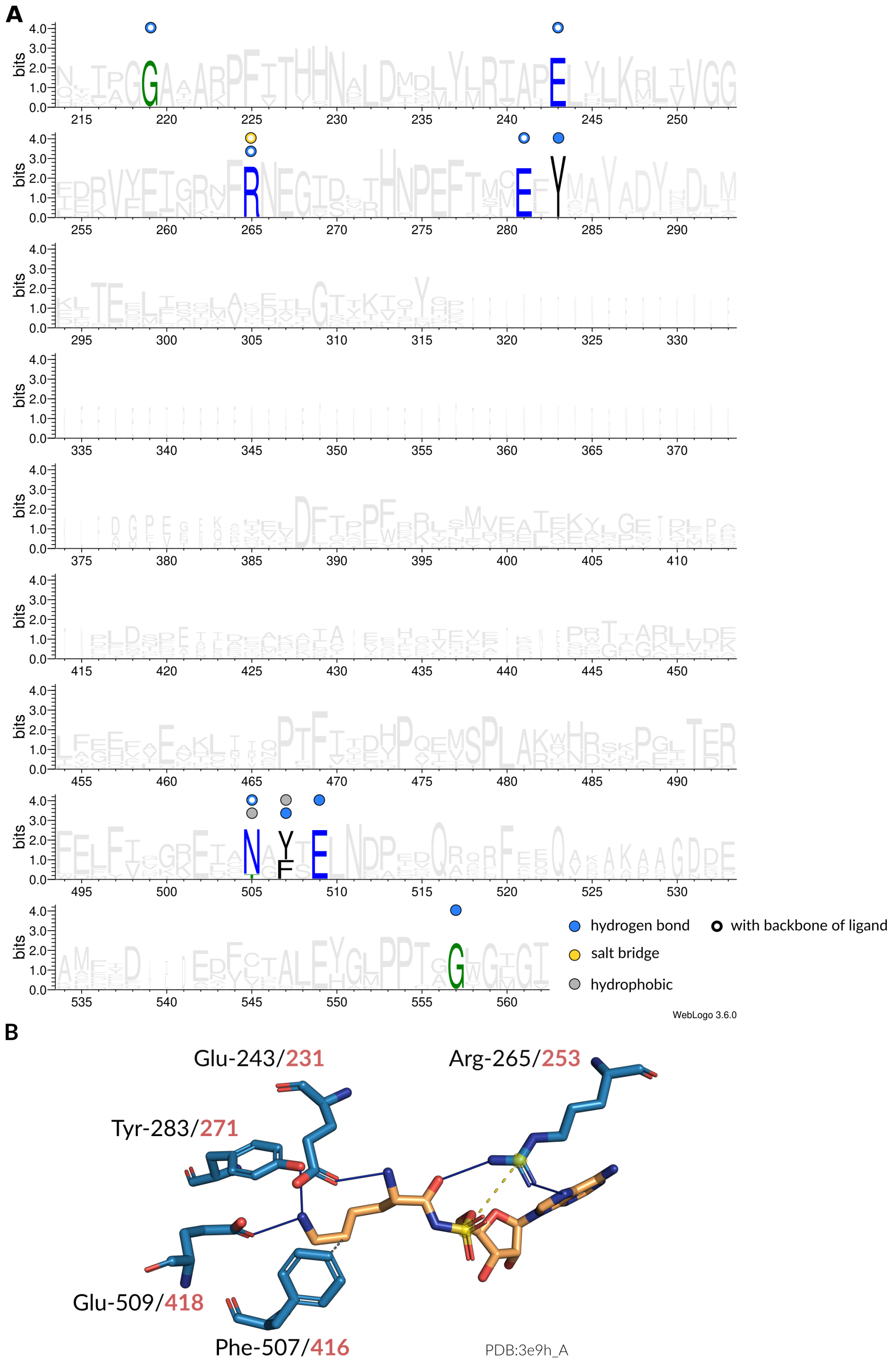

Fig. S14. Interaction patterns of lysyl-tRNA synthetase (LysRS). (**A**) Sequence logo (2) of representative sequences for LysRSs. Non-covalent interactions with the amino acid ligand occurring at certain positions are indicated by colored circles. Filled circles are interactions with the side chain atoms, while hollow circles are interactions with any of the backbone atoms of the amino acid ligand. (**B**) Depiction of interactions in the binding site (blue stick model) of an LysRS from *Geobacillus stearothermophilus* (PDB:3e9h chain A) with its ligand (orange stick model). Here, hydrogen bonds (solid blue lines), salt bridges (dashed yellow lines), and hydrophobic interactions (dashed gray lines) are established. The sequence positions of the interacting residues are given in accordance to the MSA (black) as well as the original structure (red).

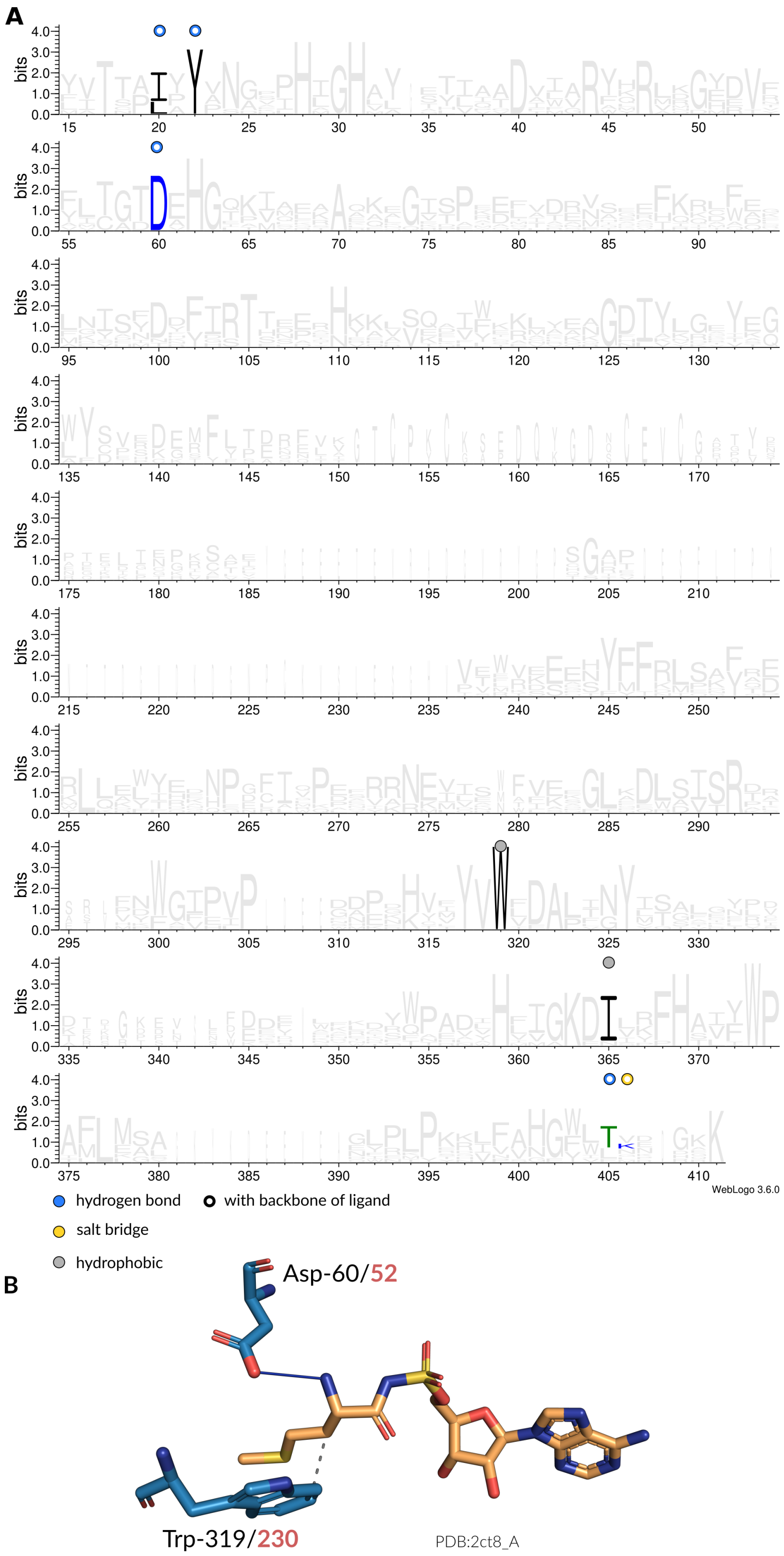

Fig. S15. Interaction patterns of methionyl-tRNA synthetase (MetRS). (**A**) Sequence logo (2) of representative sequences for MetRSs. Non-covalent interactions with the amino acid ligand occurring at certain positions are indicated by colored circles. Filled circles are interactions with the side chain atoms, while hollow circles are interactions with any of the backbone atoms of the amino acid ligand. (**B**) Depiction of interactions in the binding site (blue stick model) of an MetRS from *Aquifex aeolicus* (PDB:2ct8 chain A) with its ligand (orange stick model). Here, hydrogen bonds (solid blue lines) and hydrophobic interactions (dashed gray lines) are established. The sequence positions of the interacting residues are given in accordance to the MSA (black) as well as the original structure (red).

A

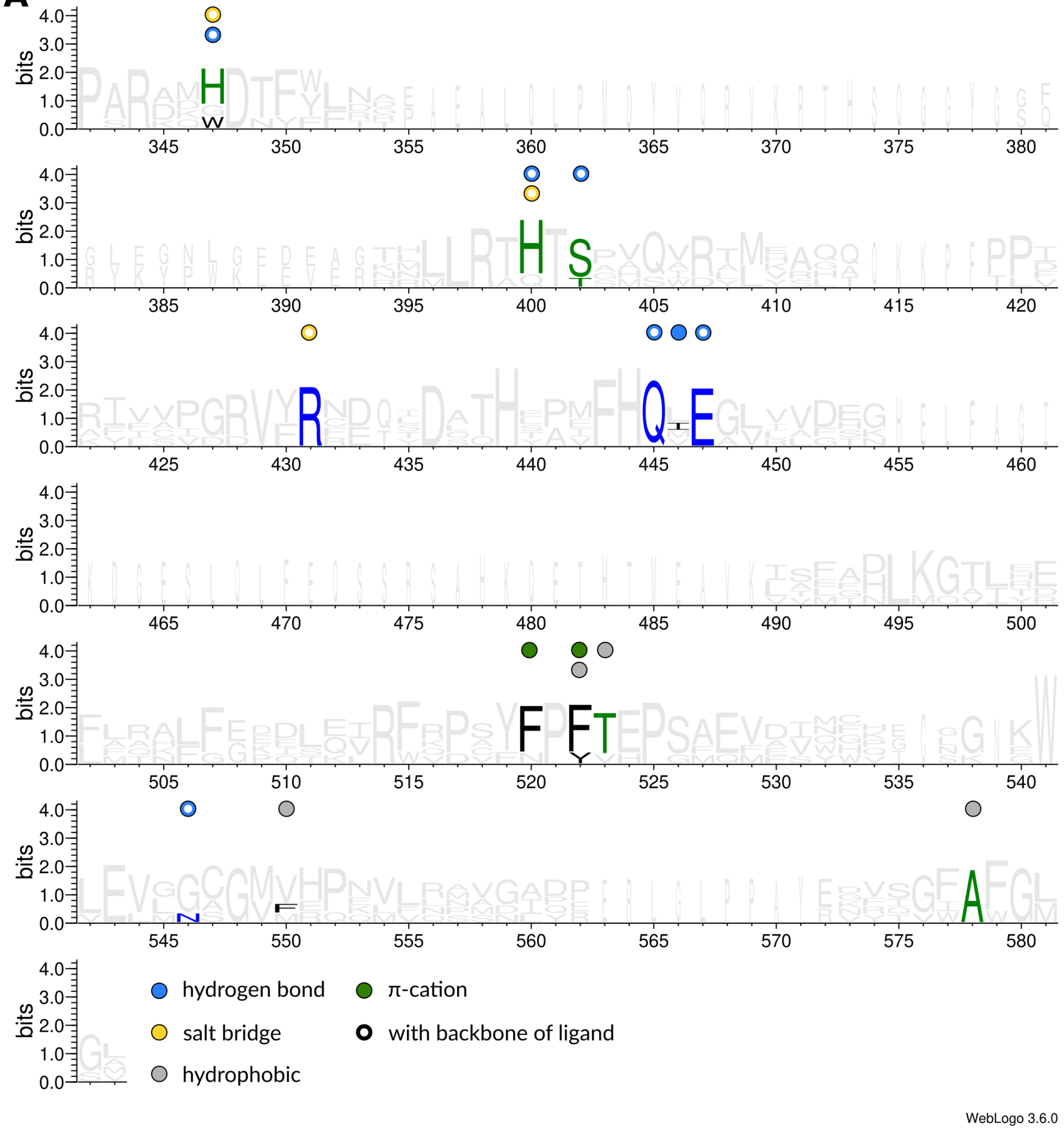

B

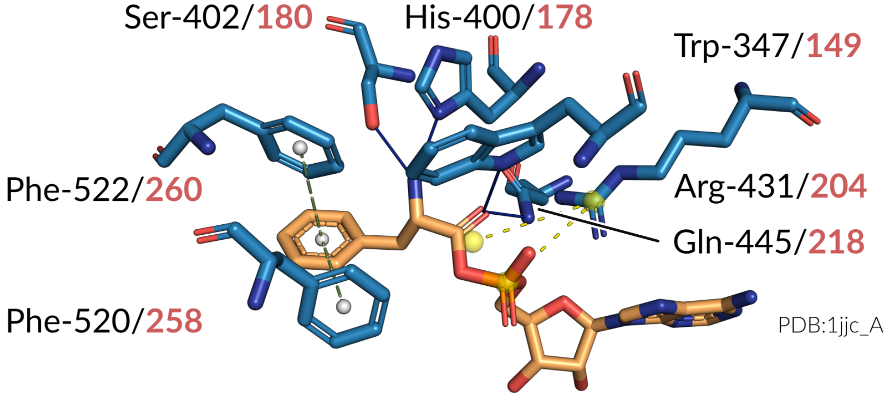

Fig. S16. Interaction patterns of phenylalanine-tRNA synthetase (PheRS). (**A**) Sequence logo (2) of representative sequences for PheRSs. Non-covalent interactions with the amino acid ligand occurring at certain positions are indicated by colored circles. Filled circles are interactions with the side chain atoms, while hollow circles are interactions with any of the backbone atoms of the amino acid ligand. (**B**) Depiction of interactions in the binding site (blue stick model) of an PheRS from *Thermus thermophilus* (PDB:1jjc chain A) with its ligand (orange stick model). Here, hydrogen bonds (solid blue lines), salt bridges (dashed yellow lines), and π-stacking interactions (dashed green lines) are established. The sequence positions of the interacting residues are given in accordance to the MSA (black) as well as the original structure (red).

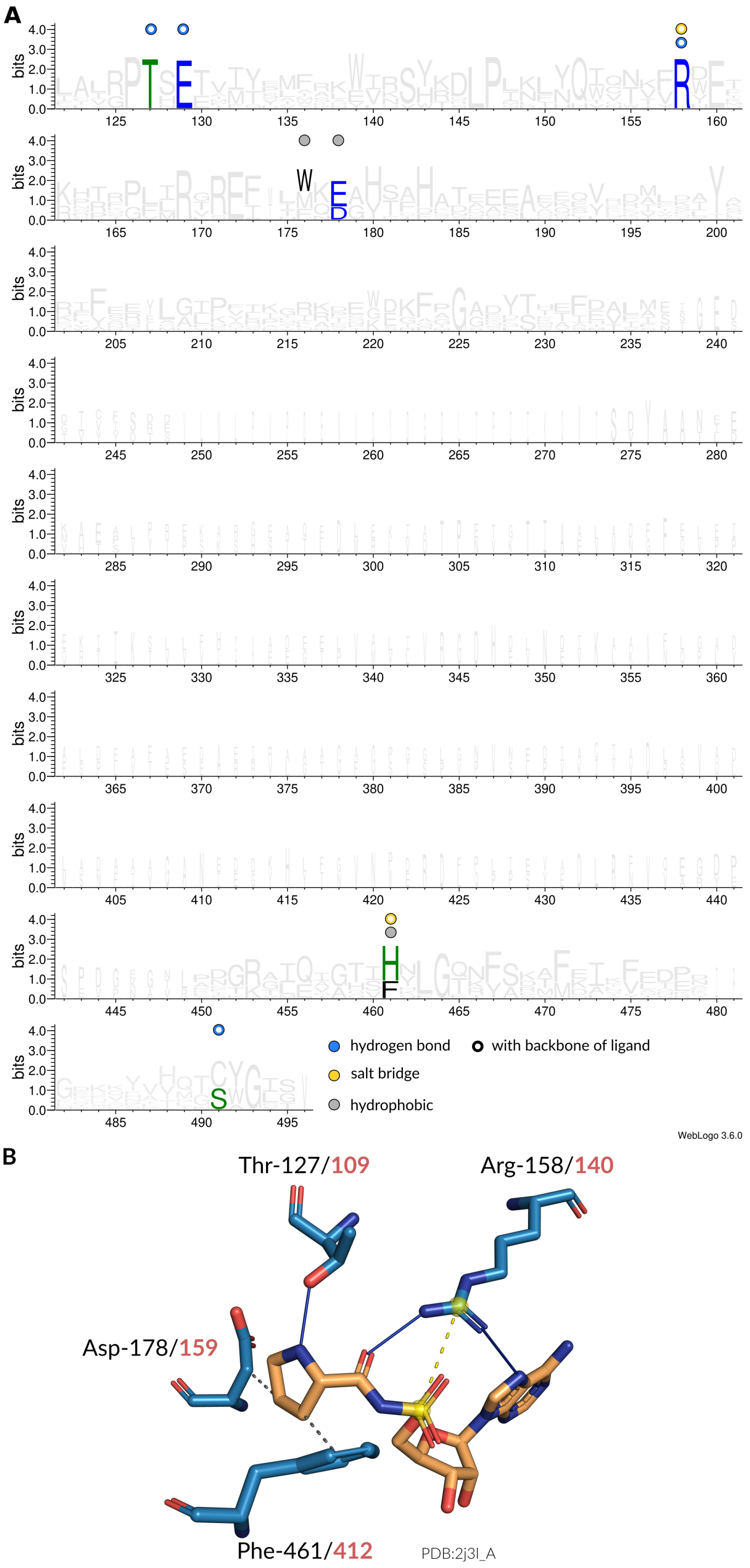

Fig. S17. Interaction patterns of prolyl-tRNA synthetase (ProRS). (**A**) Sequence logo (2) of representative sequences for ProRSs. Non-covalent interactions with the amino acid ligand occurring at certain positions are indicated by colored circles. Filled circles are interactions with the side chain atoms, while hollow circles are interactions with any of the backbone atoms of the amino acid ligand. (**B**) Depiction of interactions in the binding site (blue stick model) of an ProRS from *Enterococcus faecalis* (PDB:2j3l chain A) with its ligand (orange stick model). Here, hydrogen bonds (solid blue lines), salt bridges (dashed yellow lines), and hydrophobic interactions (dashed gray lines) are established. The sequence positions of the interacting residues are given in accordance to the MSA (black) as well as the original structure (red).

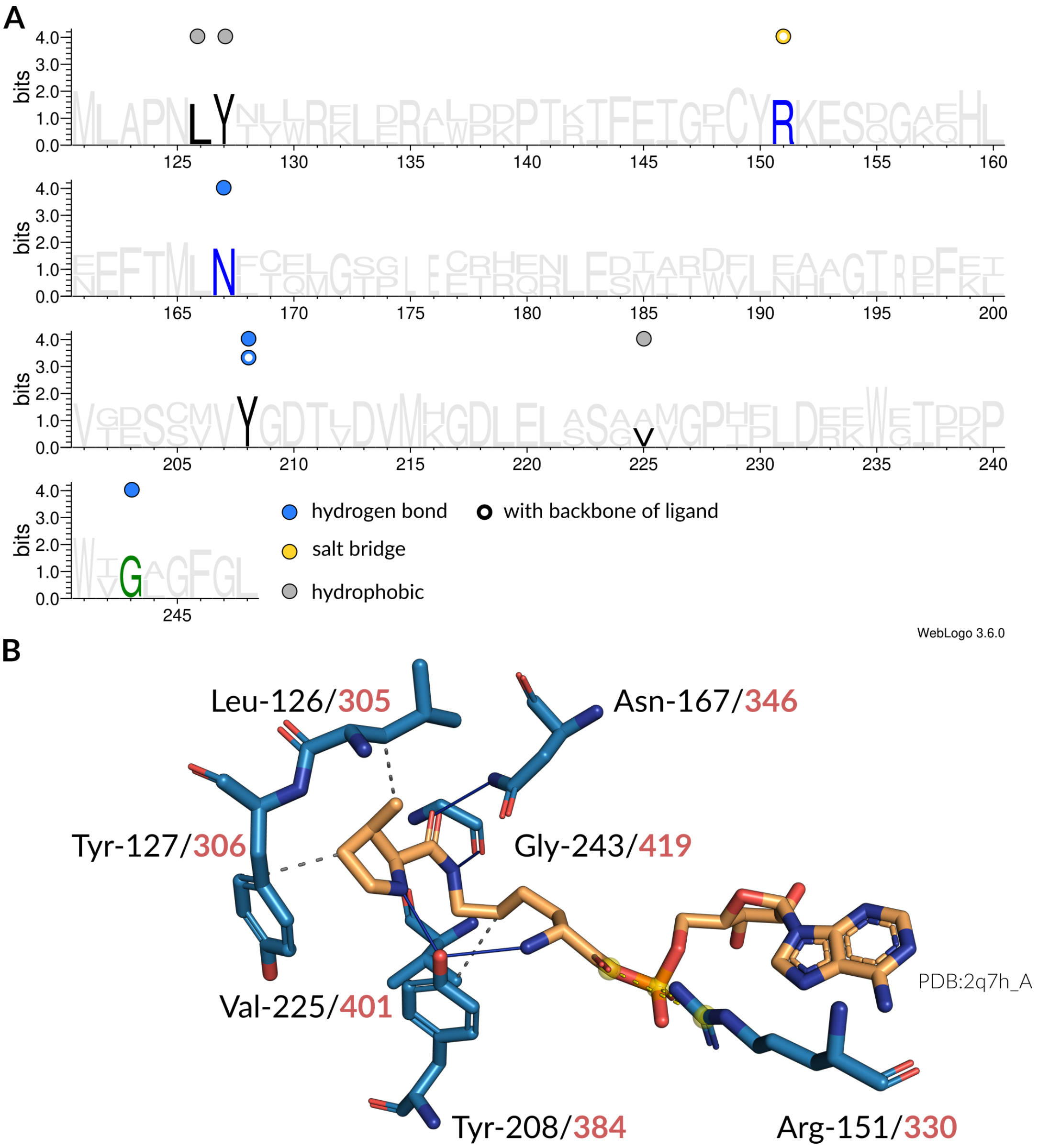

Fig. S18. Interaction patterns of pyrrolysyl-tRNA synthetase (PylRS). (**A**) Sequence logo (2) of representative sequences for PylRSs. Non-covalent interactions with the amino acid ligand occurring at certain positions are indicated by colored circles. Filled circles are interactions with the side chain atoms, while hollow circles are interactions with any of the backbone atoms of the amino acid ligand. (**B**) Depiction of interactions in the binding site (blue stick model) of an PylRS from *Methanosarcina mazei* (PDB:2q7h chain A) with its ligand (orange stick model). Here, hydrogen bonds (solid blue lines), salt bridges (dashed yellow lines), and hydrophobic interactions (dashed gray lines) are established. The sequence positions of the interacting residues are given in accordance to the MSA (black) as well as the original structure (red).

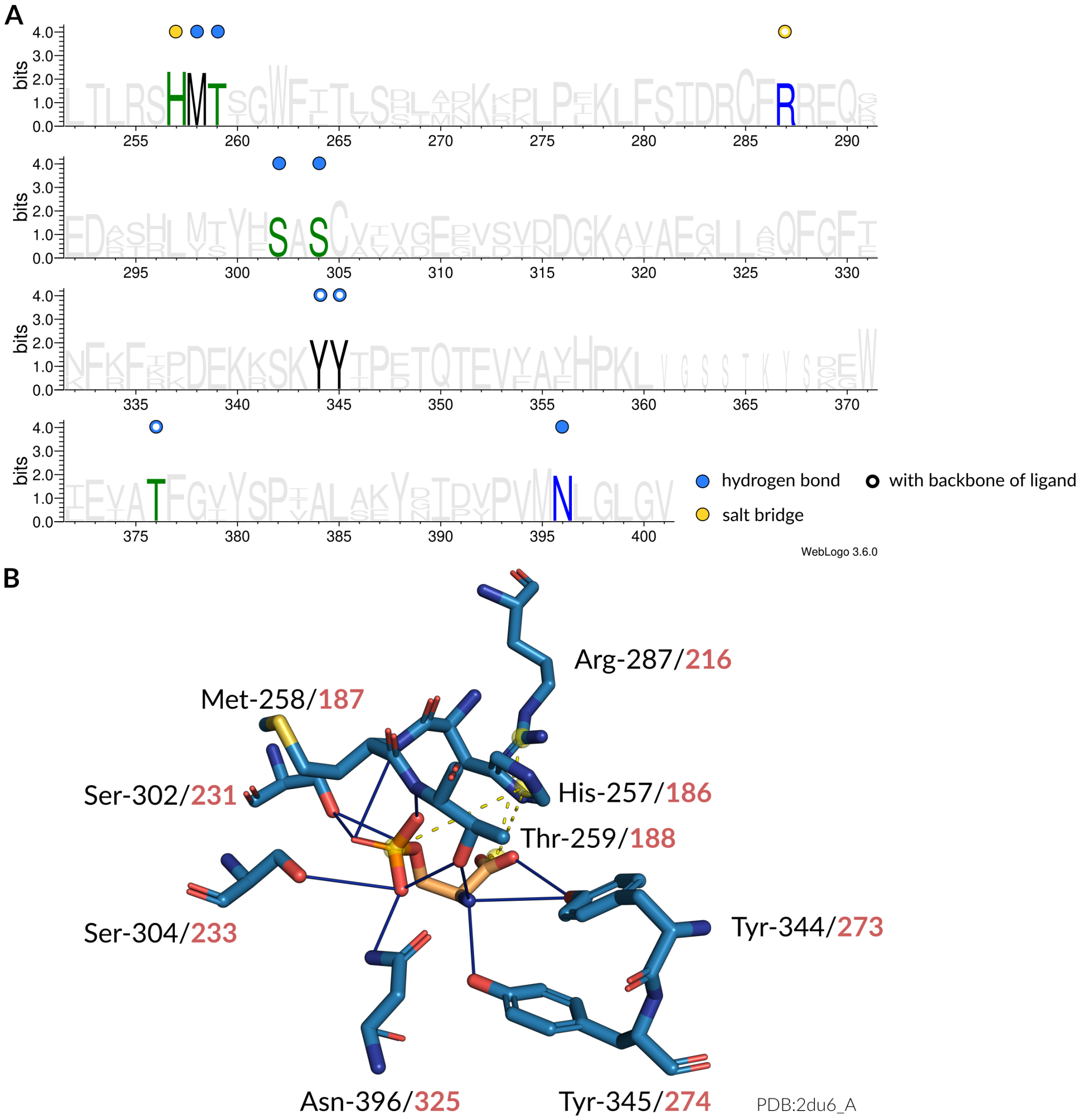

Fig. S19. Interaction patterns of phosphoseryl-tRNA synthetase (SepRS). (**A**) Sequence logo (2) of representative sequences for SepRSs. Non-covalent interactions with the amino acid ligand occurring at certain positions are indicated by colored circles. Filled circles are interactions with the side chain atoms, while hollow circles are interactions with any of the backbone atoms of the amino acid ligand. (**B**) Depiction of interactions in the binding site (blue stick model) of an SepRS from *Archaeoglobus fulgidus* (PDB:2du6 chain A) with its ligand (orange stick model). Here, hydrogen bonds (solid blue lines) and salt bridges (dashed yellow lines) are established. The sequence positions of the interacting residues are given in accordance to the MSA (black) as well as the original structure (red).

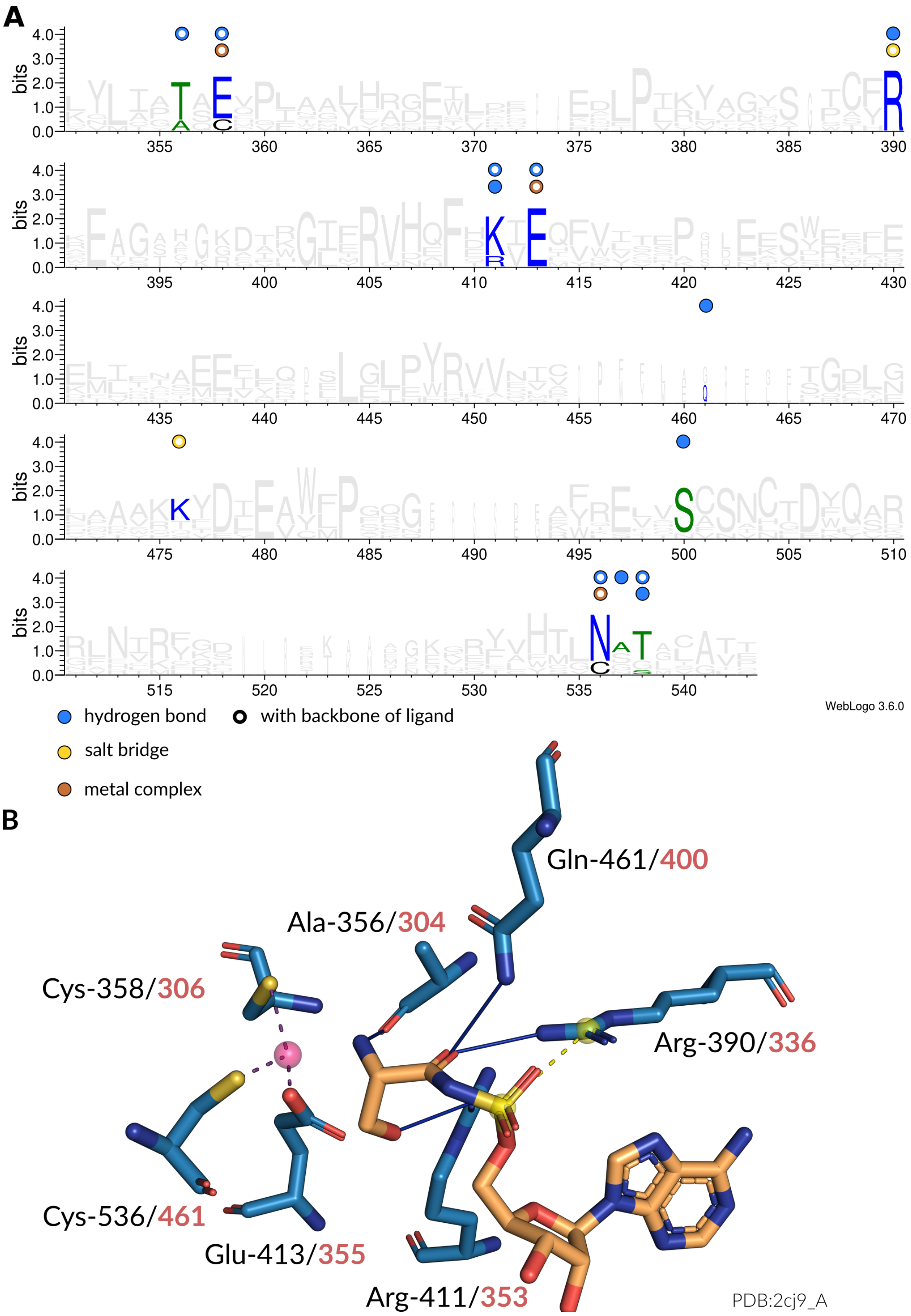

Fig. S20. Interaction patterns of seryl-tRNA synthetase (SerRS). (**A**) Sequence logo (2) of representative sequences for SerRSs. Non-covalent interactions with the amino acid ligand occurring at certain positions are indicated by colored circles. Filled circles are interactions with the side chain atoms, while hollow circles are interactions with any of the backbone atoms of the amino acid ligand. (**B**) Depiction of interactions in the binding site (blue stick model) of an SerRS from *Methanosarcina barkeri* (PDB:2cj9 chain A) with its ligand (orange stick model). Here, hydrogen bonds (solid blue lines), salt bridges (dashed yellow lines), and metal complex interactions (dashed magenta lines) are established. The sequence positions of the interacting residues are given in accordance to the MSA (black) as well as the original structure (red).

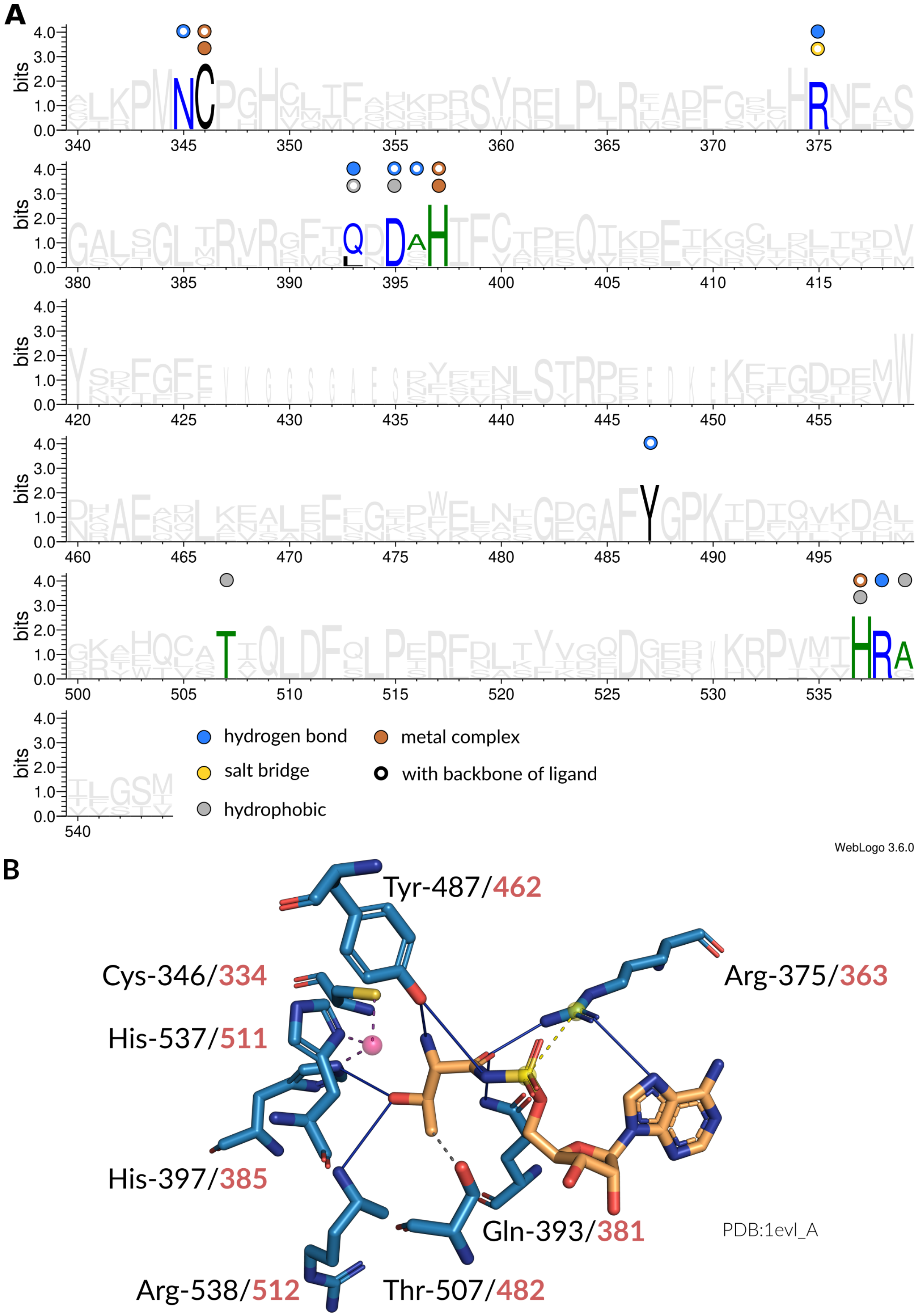

Fig. S21. Interaction patterns of threonyl-tRNA synthetase (ThrRS). (**A**) Sequence logo (2) of representative sequences for ThrRSs. Non-covalent interactions with the amino acid ligand occurring at certain positions are indicated by colored circles. Filled circles are interactions with the side chain atoms, while hollow circles are interactions with any of the backbone atoms of the amino acid ligand. (**B**) Depiction of interactions in the binding site (blue stick model) of an ThrRS from *Escherichia coli* (PDB:1evl chain A) with its ligand (orange stick model). Here, hydrogen bonds (solid blue lines), salt bridges (dashed yellow lines), metal complex interactions (dashed magenta lines), and hydrophobic interactions (dashed gray lines) are established. The sequence positions of the interacting residues are given in accordance to the MSA (black) as well as the original structure (red).

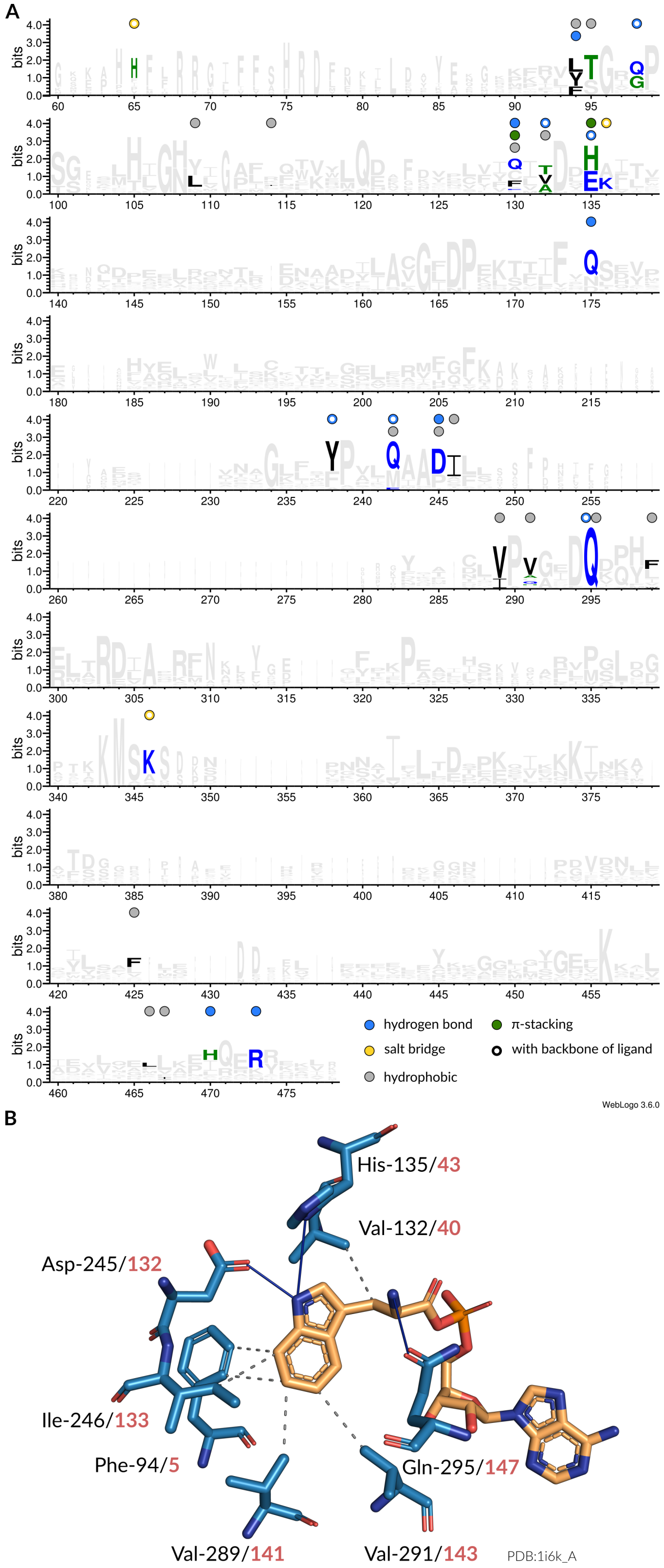

Fig. S22. Interaction patterns of tryptophanyl-tRNA synthetase (TrpRS). (**A**) Sequence logo (2) of representative sequences for TrpRSs. Non-covalent interactions with the amino acid ligand occurring at certain positions are indicated by colored circles. Filled circles are interactions with the side chain atoms, while hollow circles are interactions with any of the backbone atoms of the amino acid ligand. (**B**) Depiction of interactions in the binding site (blue stick model) of an TrpRS from *Geobacillus stearothermophilus* (PDB:1i6k chain A) with its ligand (orange stick model). Here, hydrogen bonds (solid blue lines) and hydrophobic interactions (dashed gray lines) are established. The sequence positions of the interacting residues are given in accordance to the MSA (black) as well as the original structure (red).

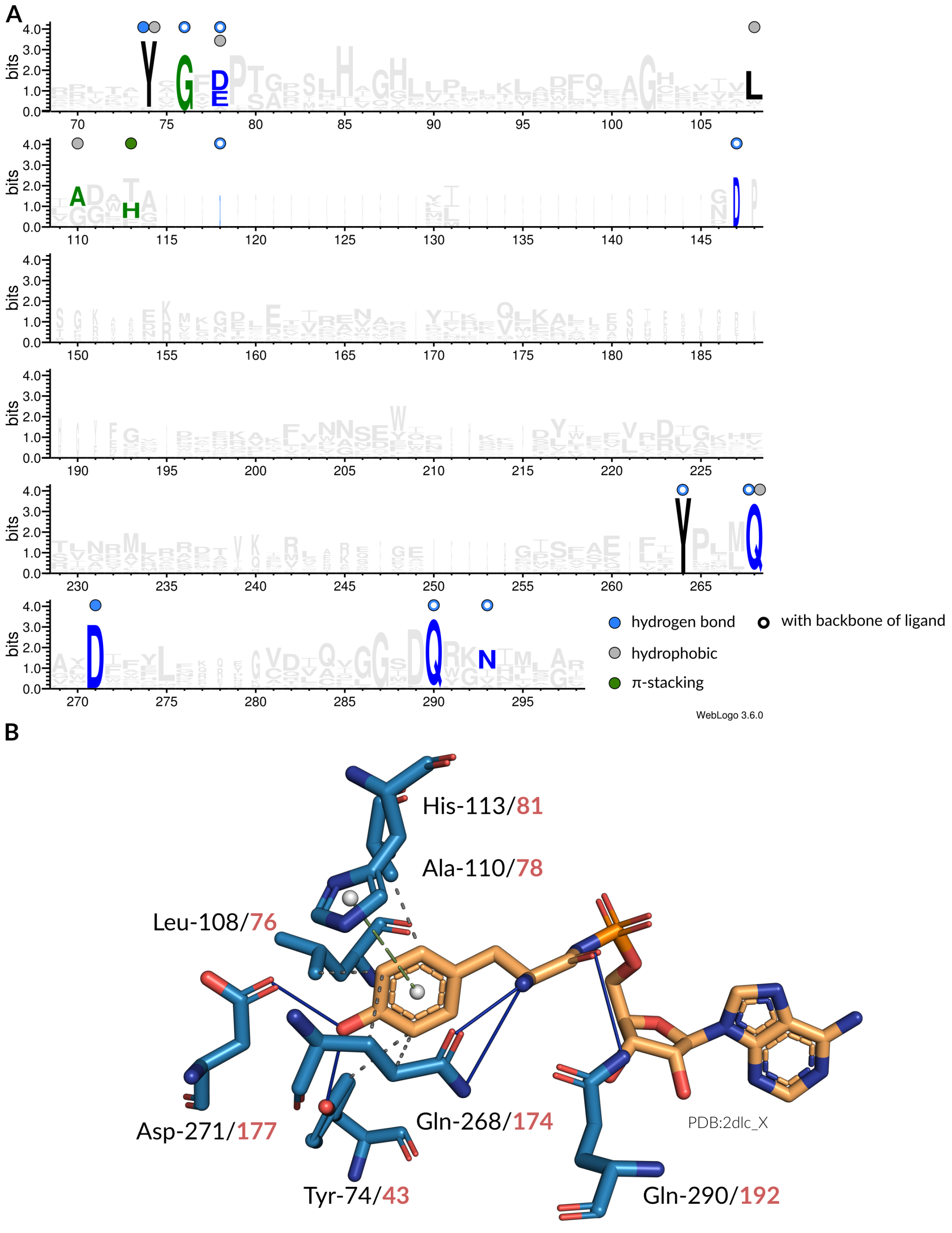

Fig. S23. Interaction patterns of tyrosyl-tRNA synthetase (TyrRS). (**A**) Sequence logo (2) of representative sequences for TyrRSs. Non-covalent interactions with the amino acid ligand occurring at certain positions are indicated by colored circles. Filled circles are interactions with the side chain atoms, while hollow circles are interactions with any of the backbone atoms of the amino acid ligand. (**B**) Depiction of interactions in the binding site (blue stick model) of an TyrRS from *Saccharomyces cerevisiae* (PDB:2dlc chain X) with its ligand (orange stick model). Here, hydrogen bonds (solid blue lines), π-stacking interactions (dashed greeen lines), and hydrophobic interactions (dashed gray lines) are established. The sequence positions of the interacting residues are given in accordance to the MSA (black) as well as the original structure (red).

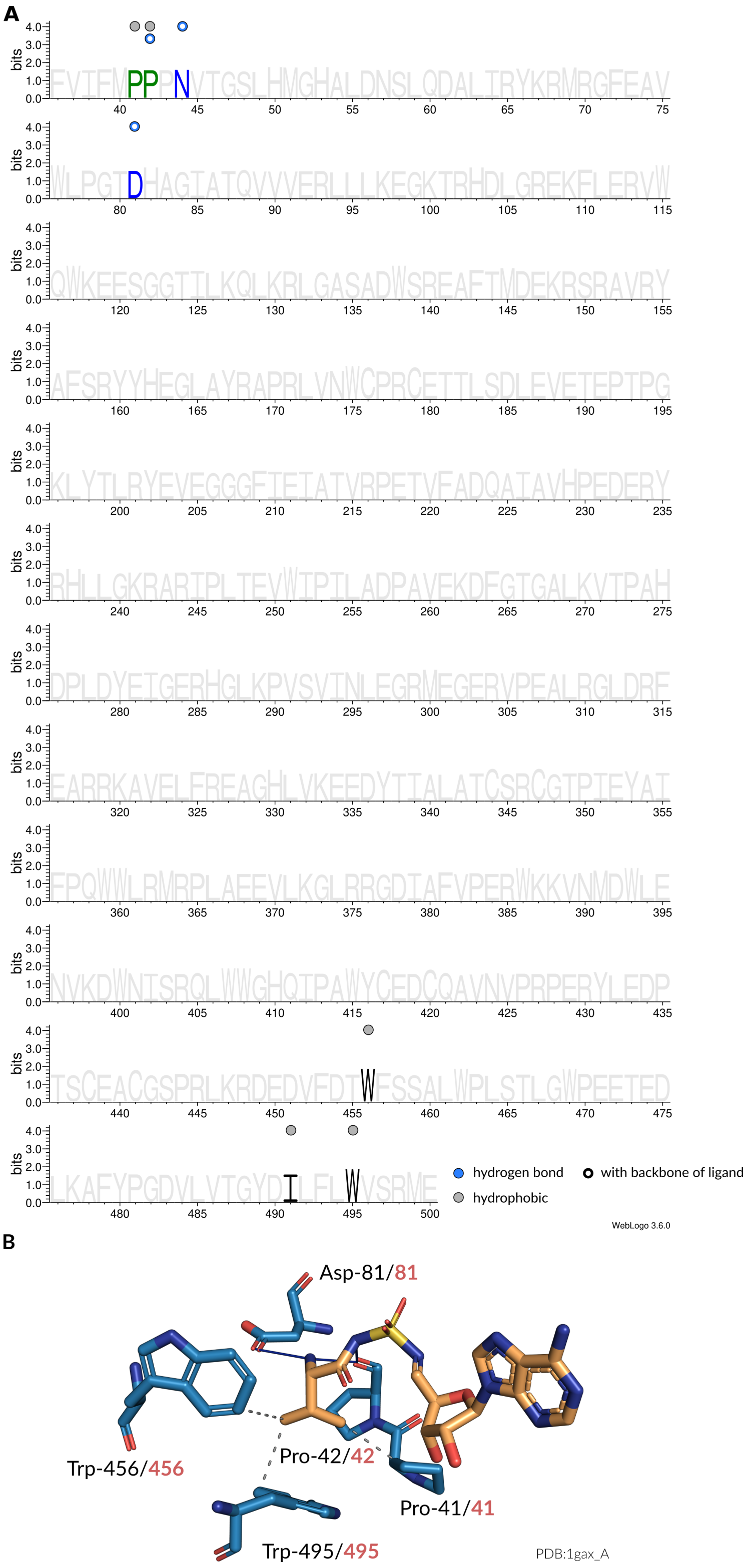

Fig. S24. Interaction patterns of valyl-tRNA synthetase (ValRS). (**A**) Sequence logo (2) of representative sequences for ValRSs. Non-covalent interactions with the amino acid ligand occurring at certain positions are indicated by colored circles. Filled circles are interactions with the side chain atoms, while hollow circles are interactions with any of the backbone atoms of the amino acid ligand. (**B**) Depiction of interactions in the binding site (blue stick model) of an ValRS from *Thermus thermophilus* (PDB:1gax chain X) with its ligand (orange stick model). Here, hydrogen bonds (solid blue lines) and hydrophobic interactions (dashed gray lines) are established. The sequence positions of the interacting residues are given in accordance to the MSA (black) as well as the original structure (red).

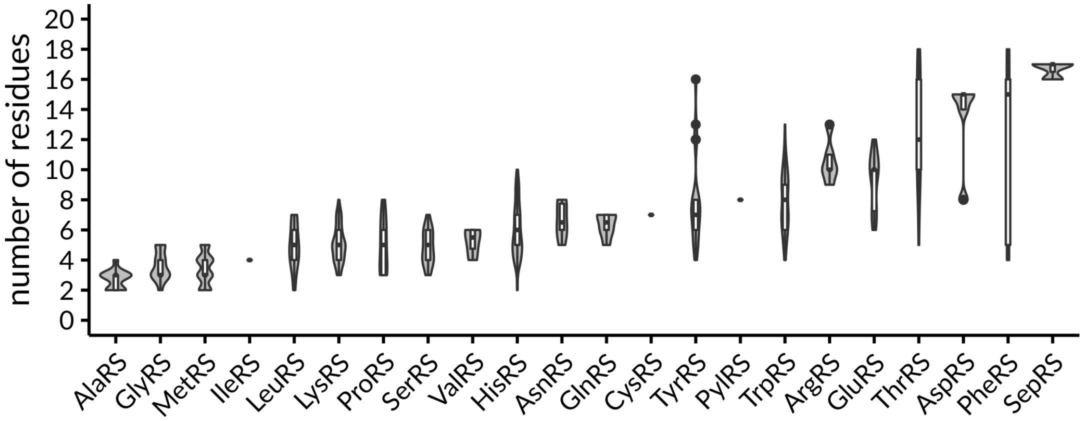

Fig. S25. The number of binding site residues involved in specificity-conferring interactions for each aaRSs. Data is sorted by ascending median from left to right.

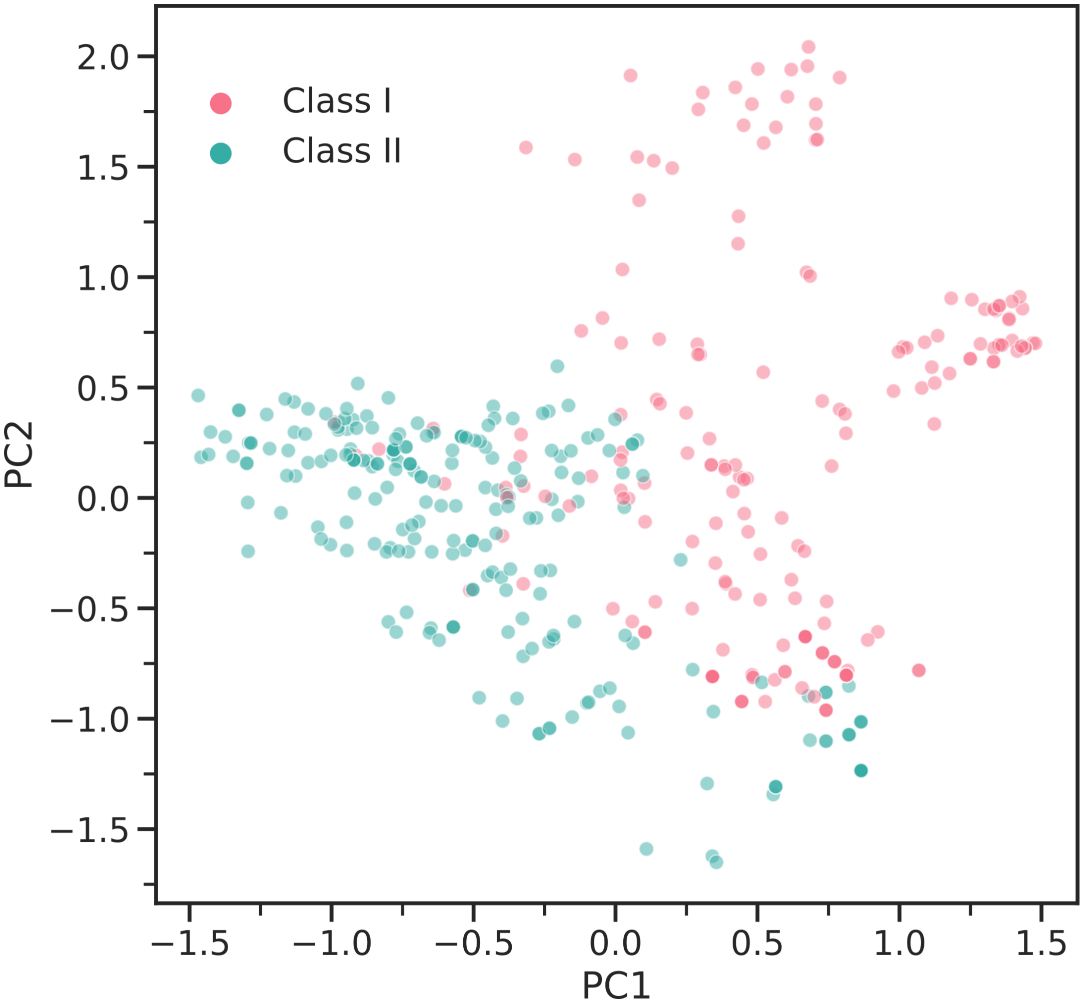

Fig. S26. Principal component analysis of interaction fingerprints colored according to the respective aaRS class. The first two components account for 9.24% and 8.44% of the covered variance, respectively. This indicated that the fingerprint representation is high-dimensional abstraction of the complex ligand recognition mechanisms in aaRSs.

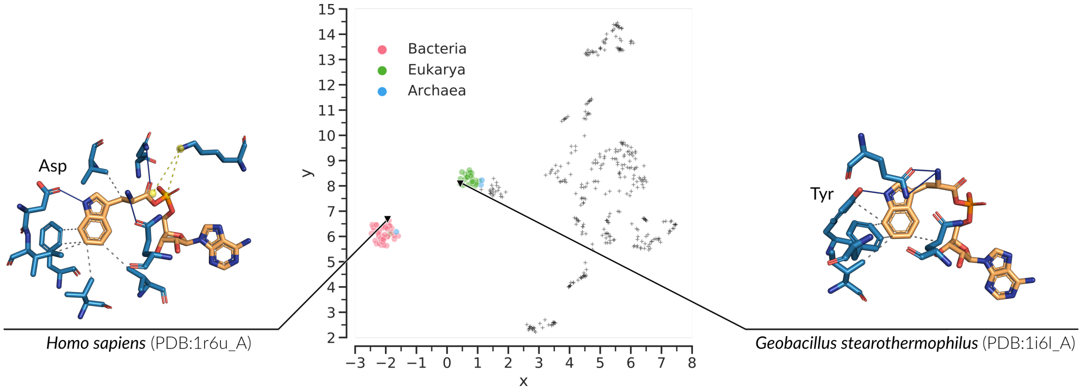

Fig. S27. Embedding space of interaction fingerprints. TrpRSs are highlighted and colored by the superkingdom of their species of origin. Two populations of TrpRSs exist, which bind their amino acid ligand in a distinct way. Two structures from both populations are shown as stick model. Hydrogen bonds (solid blue lines), salt bridges (dashed yellow lines), and hydrophobic interactions (dashed gray lines) are established. A key difference in ligand binding can be observed for a residue that binds the amino group of the indole ring. In human TrpRSs (PDB:1r6u chain A) a hydrogen bond with tyrosine is formed, while *Geobacillus stearothermophilus* (PDB:1i6l chain A) employs aspartic acid.

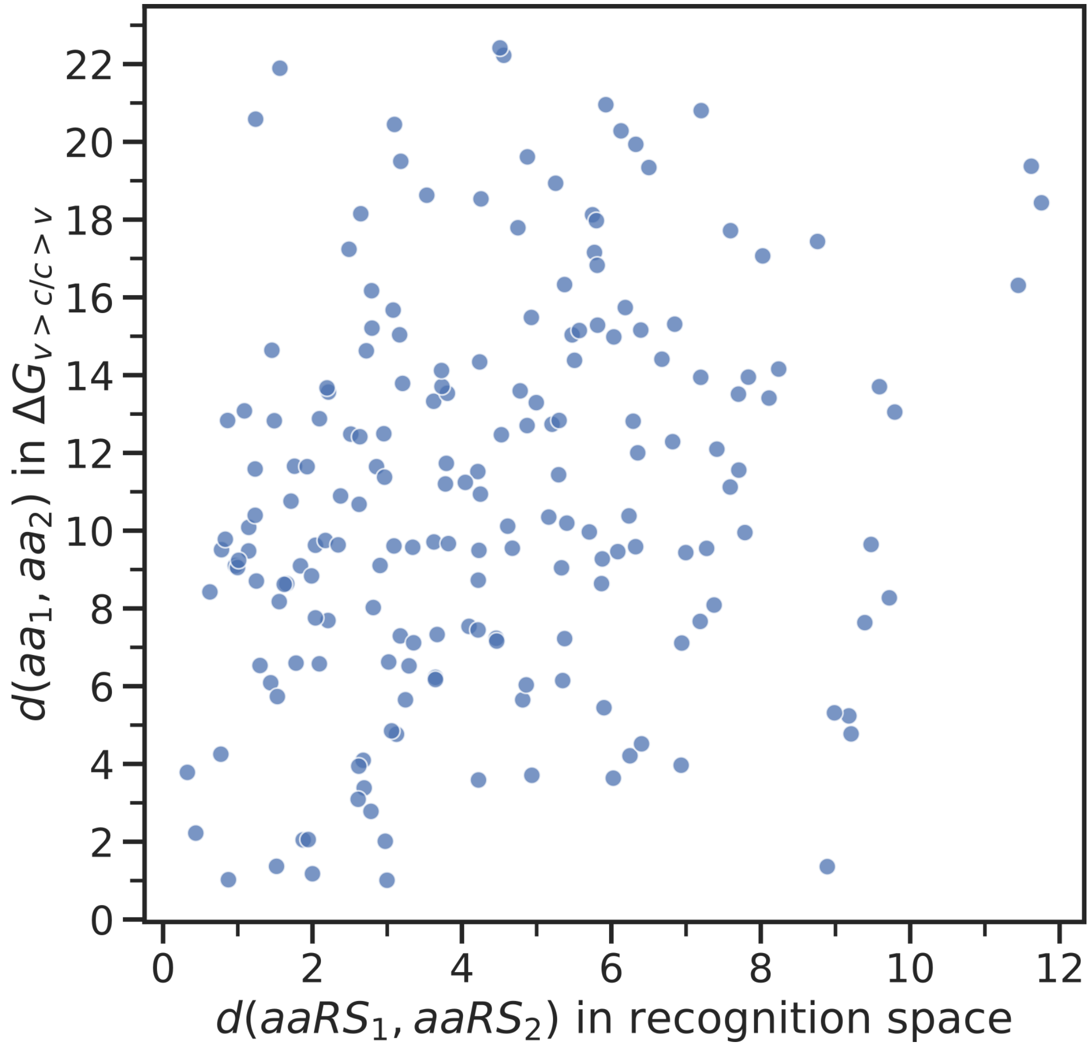

Fig. S28. Phase transfer free energies of amino acid side chains (3, 4) from water ($\Delta G_{w>c}$) and vapor ($\Delta G_{w>c}$) to cyclohexane compared to the recognition space analysis from this study. Each data point represents the euclidean distance between every combination of two amino acids in the phase transfer diagram given in Carter and Wills (5) against the euclidean distance in the recognition space proposed in this study. Spearman's rank correlation is $\rho = 0.2564$ with p<0.01.

References

1. F Kaiser, et al., Backbone Brackets and Arginine Tweezers delineate Class I and Class II aminoacyl tRNA synthetases. *PLoS Comput. Biol.* **14**, e1006101 (2018).
2. GE Crooks, G Hon, JM Chandonia, SE Brenner, WebLogo: a sequence logo generator. *Genome Res.* **14**, 1188–1190 (2004).
3. CW Carter, R Wolfenden, tRNA acceptor stem and anticodon bases form independent codes related to protein folding. *Proc. Natl. Acad. Sci. U.S.A.* **112**, 7489–7494 (2015).
4. R Wolfenden, CA Lewis, Y Yuan, CW Carter, Temperature dependence of amino acid hydrophobicities. *Proc. Natl. Acad. Sci. U.S.A.* **112**, 7484–7488 (2015).
5. C W. Carter, P Wills, *Did Gene Expression Co-evolve with Gene Replication?* (Springer International Publishing), pp. 293–313 (2018).
